# Cytokine-induced killer cells/dendritic cells-cytokine induced killer cells immunotherapy combined with chemotherapy for treatment of colorectal cancer in China: a meta-analysis of 29 trials involving 2,610 patients

**DOI:** 10.18632/oncotarget.16665

**Published:** 2017-03-29

**Authors:** Lei Zhang, Ying Mu, Anqi Zhang, Jiaping Xie, Shuangfeng Chen, Fang Xu, Weihua Wang, Yingxin Zhang, Shaoda Ren, Changhui Zhou

**Affiliations:** ^1^ Institute of Hematopathy, Henan Provincial People's Hospital, Zhengzhou, Henan Province, China; ^2^ Department of Gastroenterology, Liaocheng People's Hospital, Liaocheng Clinical School of Taishan Medical University, Liaocheng, Shandong Province, China; ^3^ Department of Central Laboratory, Liaocheng People's Hospital, Liaocheng Clinical School of Taishan Medical University, Liaocheng, Shandong Province, China; ^4^ Institute of Medical and Pharmaceutical Sciences, Zhengzhou University, Zhengzhou, Henan Province, China

**Keywords:** cytokine-induced killer cells, dendritic cells, colorectal cancer, immunotherapy, meta-analysis

## Abstract

**Purpose:**

To systematically evaluate the efficacy and safety of Cytokine-induced killer cells/dendritic cells-cytokine induced killer cells (CIK/DC-CIK) immunotherapy in treating advanced colorectal cancer (CRC) patients.

**Results:**

29 trials including 2,610 CRC patients were evolved. Compared with chemotherapy alone, the combination of chemotherapy with CIK/DC-CIK immunotherapy significantly prolonged the overall survival rate (OS) and disease-free survival rate (DFS) (1–5 year OS, *P* < 0.01; 1-, 2-, 3- and 5-year DFS, *P* < 0.01). The combined therapy also improved patients’ overall response, disease control rate and life quality (*P* < 0.05). After immunotherapy, lymphocyte subsets percentages of CD3^+^, CD3^−^CD56^+^, CD3^+^CD56^+^ and CD16^+^CD56^+^ (*P* < 0.01) and cytokines levels of IL-2 and IFN-γ (*P* < 0.05) were increased, while CD4^+^, CD8^+^ and CD4^+^CD25^+^ and IL-6 and TNF-α did not show significant change (*P* > 0.05).

**Materials and Methods:**

Clinical trials reporting response or safety of CIK/DC-CIK immunotherapy treating advanced CRC patients and published before September 2016 were searched in Cochrane Library, EMBASE, PubMed, Wanfang and CNKI database. Research quality and heterogeneity were evaluated before analysis. Pooled analyses were performed using random or fixed-effect models.

**Conclusions:**

The combination of CIK/DC-CIK immunotherapy and chemotherapy prolong CRC patients’ survival time, enhanced patients’ immune function and alleviates the adverse effects caused by chemotherapy.

## INTRODUCTION

Colorectal cancer (CRC) is currently the third common malignant tumor [1, 2]. In recent years, CRC incidence in newly developed or economically transitional countries have been significantly raised, and the 5-year survival rate of IV stage CRC patients is lower than 10% [2]. Surgery, radiotherapy and chemotherapy are the most widely used therapeutic methods for CRC [2], but their curative effects on CRC patients were poor. Researchers found these methods were not able to thoroughly remove small lesions and metastatic cells, which raises the probability of cancer recurrence [3]. Application of these treatments is also limited by drug resistance and adverse effects [3, 4]. Therefore, a more effective and safer therapeutic method is urgently required.

Immunotherapy is an emerging approach for CRC treatment, especially adoptive cell therapy by cytokine-induced killer cells (CIK) [5], tumor-infiltrating lymphocytes [6] and other immune cells [7, 8]. CIK cells, which consist primarily of the CD3^+^CD56^+^ subset, are induced by IFN-γ, anti-CD3 monoclonal antibodies and IL-2 *in vitro* [4]. Compared with other immune cells, CIK cells have greater proliferative capability, broader anti-tumor spectrum and stronger anti-tumor ability [3, 9]. CIK cells’ tumoricidal ability relies on inducing tumor cell apoptosis through direct contact and secretion of cytokines such as IL-2, TNF-a and IFN-γ [10], and the cytotoxicity of CIK cells is not affected by immune inhibitors such as CsA and FK506 [11]. Dendritic cells (DC) are the most potent antigen-presenting cells [12]. Studies have shown that DC play an important role in CIK activation, proliferation, phenotype expression and cytokine secretion by direct contact and secreted IL-12, IFN-γ, and TNF-a [13–16]. Co-culture of DC and CIK showed increased levels of cytokines such as IL-2, IFN-γ, TNF-a, and IL-12, but downregulated negative regulatory factors including TGF-β and IL-10. Upon DC and CIK co-culture, the proportion of CD3^+^CD56^+^ cells, which are the main effector cells enhancing CIK cytotoxicity, were increased; whereas CD4^+^CD25^+^ regulatory T cells, which inhibit CIK anti-tumor activity, where decreased [13–15]. On the other hand, CIK cells promote DC maturation and expression of co-stimulatory molecules such as CD40, CD80 and CD86 [3, 13], and the combination of DC and CIK provides a remarkably increased cytotoxic activity [17].

Immunotherapy using CIK/DC-CIK was widely reported to be effective in treating various malignancies, without causing serious adverse reactions [18–28]. In CRC treatment, CIK/DC-CIK immunotherapy combined with chemotherapy had better therapeutic effects than treated by chemotherapy alone (Supplementary Table 1) [5, 18, 29–55]. To investigate the efficacy and safety of CIK/DC-CIK immunotherapy for CRC and thus provide scientific evidence for future clinical trials, we performed a systematic review and meta-analysis of published literature on recent CIK/DC-CIK and chemotherapy combined clinical trials for CRC. This review evaluates the patient survival, clinical responses, safety, and immune functions in this combined therapeutic method.

## RESULTS

### Search results

**Figure 1:**
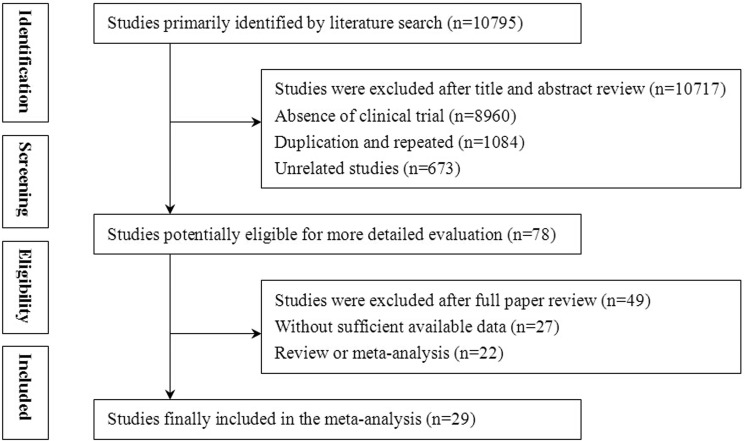
Flow diagram of the selection process

In our initial retrieve, 10,795 articles were identified, but 10,717 were excluded for lacking of clinical trial (*n* = 8,960), duplication (*n* = 1084), and unrelated studies (*n* = 673). After a detailed assessment of full texts, 27 papers with insufficient data and 22 reviews or meta-analyses were excluded. Finally, 29 trials that included a total of 2,610 patients were eligible for inclusion in this meta-analysis (Figure 1).

### Patient characteristics

All of the involved trials were conducted in mainland China. In total, 1300 patients were treated by CIK/DC-CIK in combination with chemotherapy, while 1310 patients were treatment by chemotherapy alone. Detailed clinical information on the trials is presented in Supplementary Table 1. DC and CIK cells used in the 29 trials were all obtained from autologous peripheral blood. DC-CIK immunotherapy was applied in 20 trials, whereas only CIK cells were used in the other 9 trials. Tumor size and injection modes were not analyzed in this article because of insufficient data.

### Quality assessment

**Figure 2:**
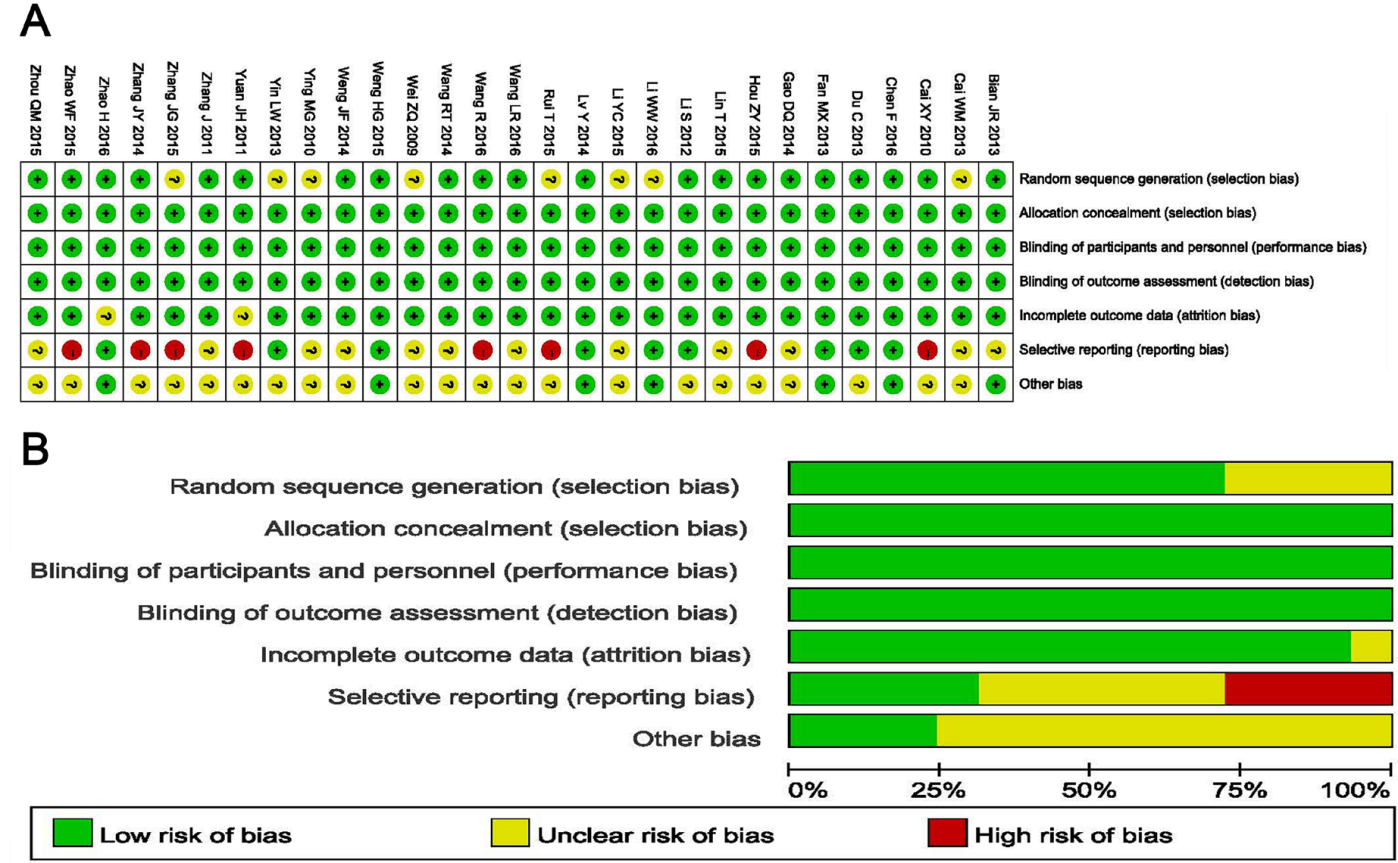
(**A**) Risk of bias summary: review of authors’ judgments about each risk of bias item for included studies. (**B**) Risk of bias graph: review of authors’ judgments about each risk of bias item presented as percentages across all included studies.

Bias risk assessment is shown in Figure 2A and 2B. According to the assessment, quality of involved studies was moderate or high. 21 studies had low bias risk, and the remaining 8 studies lacked a clear description of randomization process. The allocation, performance and detection risks were low. 2 studies were regarded as an unclear risk due to the absence of follow-up. 12 trials were considered as unclear risk owing to selective reporting, while the other 8 studies were considered as high risk because of the lack of primary outcome data.

### Prognosis evaluation

**Figure 3:**
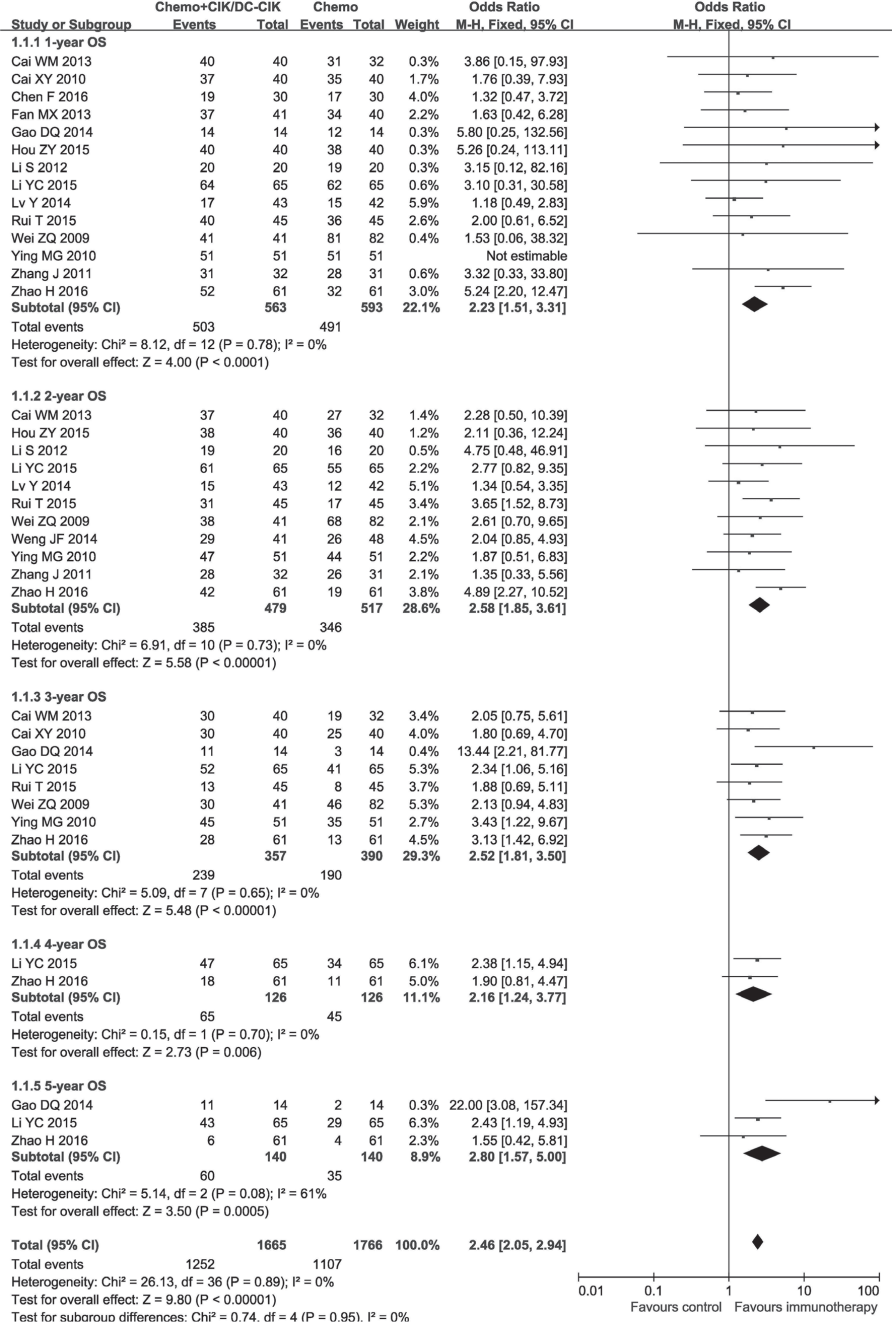
Forest plot of the comparison of overall survival (OS) CI, confidence interval; Chemo, chemotherapy; CIK/DC-CIK, CIK/DC-CIK immunotherapy. The fixed-effects meta-analysis model (Mantel–Haenszel method) was used.

**Figure 4:**
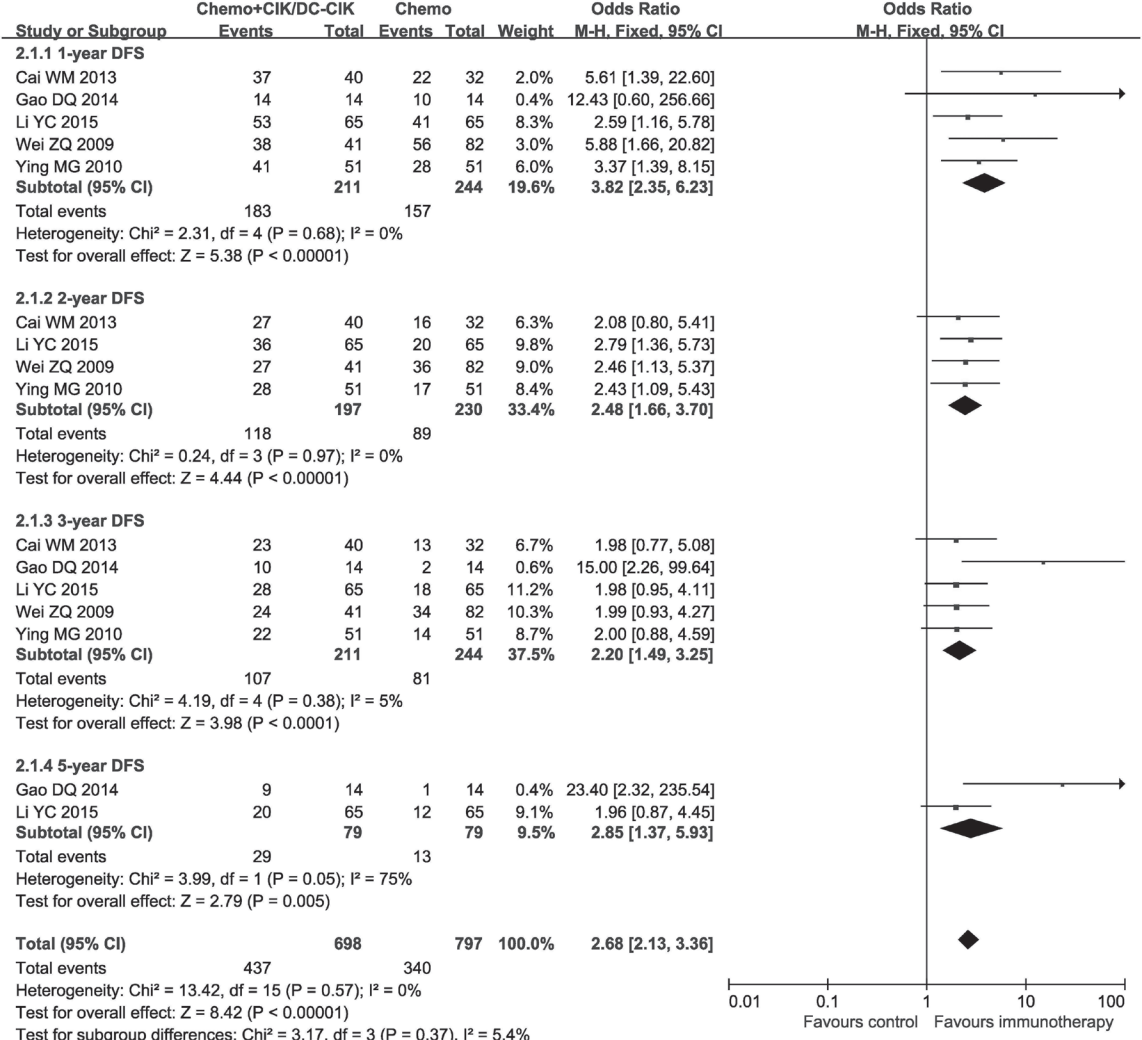
Forest plot of the comparison of disease free survival (DFS) CI, confidence interval; Chemo, chemotherapy; CIK/DC-CIK, CIK/DC-CIK immunotherapy. The fixed-effects meta-analysis model (Mantel–Haenszel method) was used.

In the 29 studies, patients treated by combined therapy (combined group) have higher overall survival rate (OS) and disease free survival rate (DFS) than those treated by chemotherapy alone (chemo-alone group) (Figures 3 and 4, 1-year OS: OR = 2.23, CI = 1.51–3.31, *P* < 0.0001; 2-year OS: OR = 2.58, CI = 1.85–3.61, *P*< 0.00001; 3-year OS: OR = 2.52, CI = 1.81–3.50, *P* < 0.00001; 4-year OS: OR = 2.16, CI = 1.24–3.77, *P* = 0.006; 5-year OS: OR = 2.80, CI = 1.57–5.00, *P* = 0.0005; 1-year DFS: OR = 3.82, CI = 2.35–6.23, *P* < 0.00001; 2-year DFS: OR = 2.48, CI = 1.66–3.70, *P* < 0.00001; 3-year DFS: OR = 2.20, CI = 1.49–3.25, *P* < 0.0001; 5-year DFS: OR = 2.85, CI = 1.37–5.93, *P* = 0.005). Fixed-effects model were applied in this analysis considering slightly significant heterogeneity.

### Efficacy assessments

**Figure 5:**
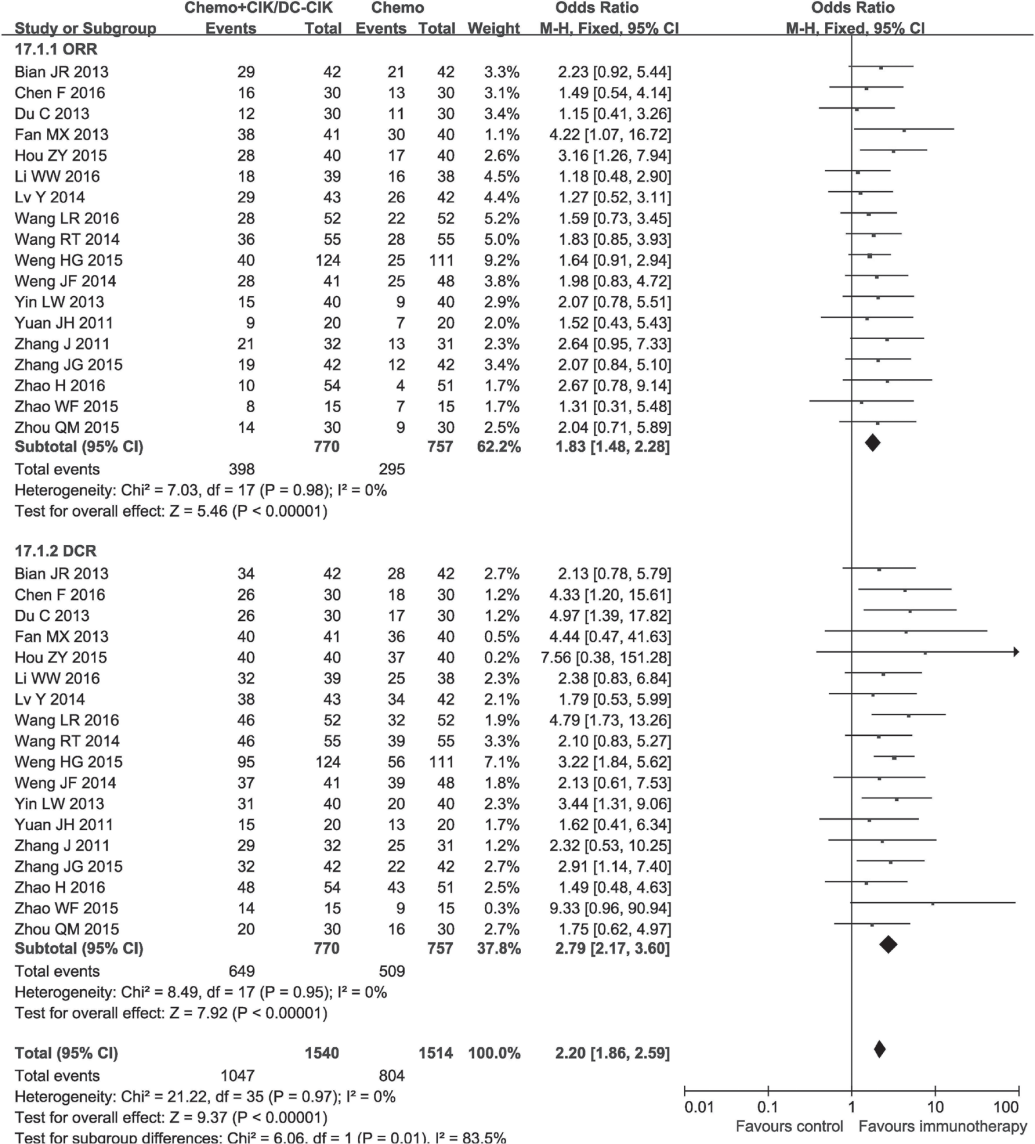
Forest plot of the comparison of overall response rate (ORR) and disease control rate (DCR) CI, confidence interval; Chemo, chemotherapy; CIK/DC-CIK, CIK/DC-CIK immunotherapy. The fixed-effects meta-analysis model (Mantel– Haenszel method) was used.

Pooled analysis indicated patients in combined group had significantly higher complete response rates (CR), partial response rates (PR), overall response rate (ORR) and disease control rate (DCR) (CR: OR = 1.96, CI = 1.22−3.15, *P* = 0.005; PR: OR = 1.53, CI = 1.22−1.92, *P* = 0.0003; ORR: OR = 1.83, CI = 1.48−2.28, *P* < 0.00001; DCR: OR = 2.79, CI = 2.17−3.60, *P* < 0.00001) and significantly lower progressive disease rates (PD) (OR = 0.36, CI = 0.27−0.47, *P* < 0.00001), whereas the stable disease rates (SD) did not show significant difference from chemo-alone group (OR = 1.25, CI = 0.98−1.59, *P* = 0.07). Fixed-effect models were used to analyze the OR rate because of low heterogeneity (Figure 5, Table 1 and Supplementary Figure 1).

**Table 1:** Comparison of CR, PR, SD, PD, ORR and DCR between the chemotherapy+CIK/DC-CIK and chemotherapy alone groups

Parameter	Chemo+CIK/DC-CIK	Chemo	Analysis method	Heterogeneity		Odds Ratio (OR)	95% CI	*P*-value
	No. patients (*n*)	No. patients (*n*)		*I*^2^ (%)	*P*-value			
CR	689	669	Fixed	0	0.94	1.96	1.22 to 3.15	0.005
PR	689	669	Fixed	0	0.99	1.53	1.22 to 1.92	0.0003
SD	689	669	Fixed	42	0.04	1.25	0.98 to 1.59	0.07
PD	689	669	Fixed	0	0.92	0.36	0.27 to 0.47	< 0.00001
ORR	770	757	Fixed	0	0.98	1.83	1.48 to 2.28	< 0.00001
DCR	770	757	Fixed	0	0.95	2.79	2.17 to 3.60	< 0.00001

CR: complete response rates; PR: partial response rates; SD: stable disease rates; PD: progressive disease rates; ORR: overall response rate; DCR: disease control rate. Chemo: chemotherapy.

### Quality of life (QOL) assessment

**Figure 6:**
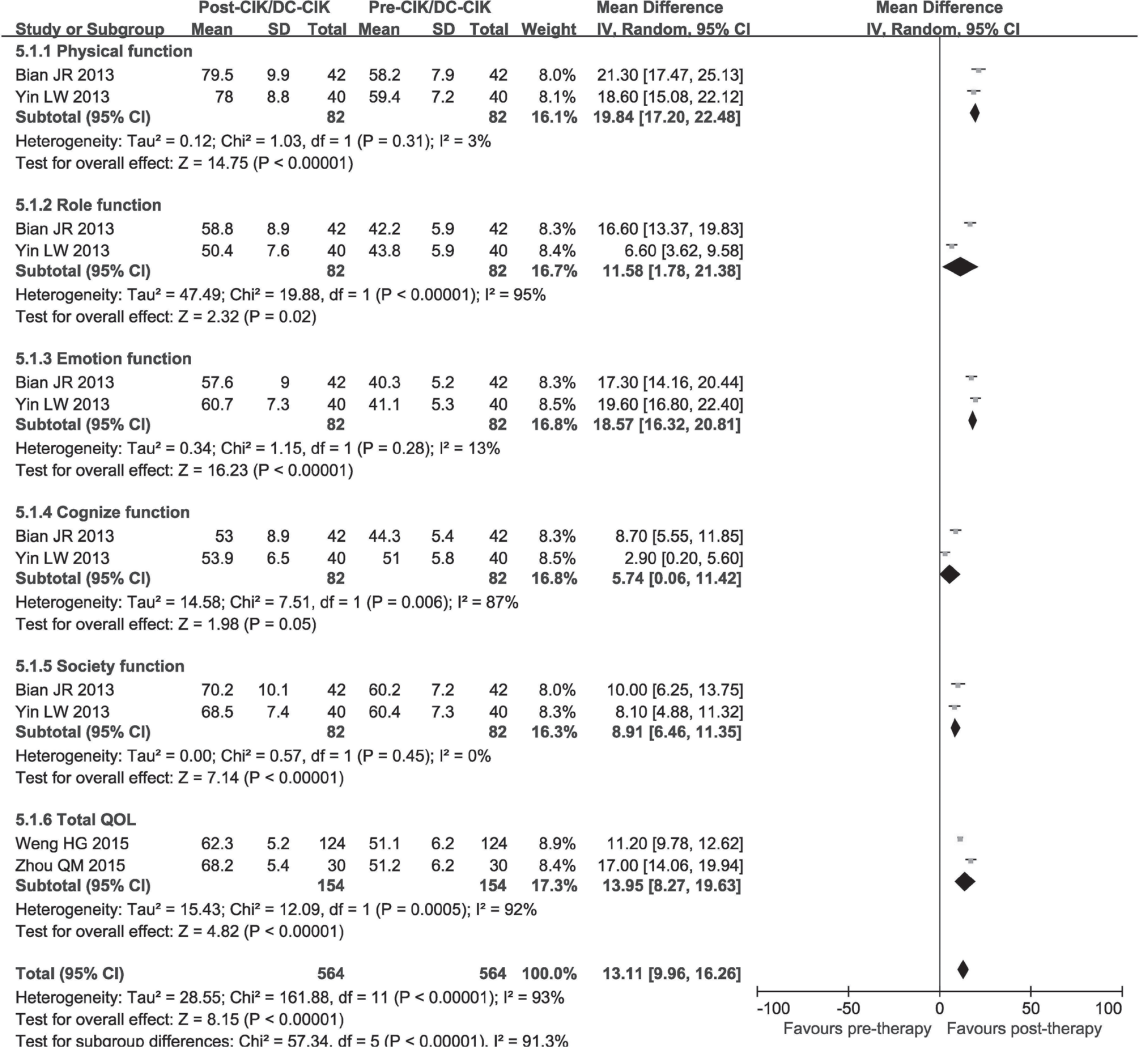
Forest plot of the comparison of QQL CI, confidence interval; Chemo, chemotherapy; CIK/DC-CIK, CIK/DC-CIK immunotherapy. The random effects meta-analysis model (Mantel–Haenszel method) was used.

QOL was evaluated before and after therapy and analyzed with random-effect models. QOL assessment shows that in combined group, patients after CIK/DC-CIK immunotherapy showed significantly improved physical function (OR = 19.84, CI = 17.20−22.48, *P* < 0.00001), emotion function (OR = 18.57, CI = 16.32−20.81, *P* < 0.00001), cognize function (OR = 5.74, CI = 0.06−11.42, *P* = 0.05), society function (OR = 8.91, CI = 6.46−11.35, *P* < 0.00001) and but role function (OR = 11.58, CI = 1.78−21.38, *P* = 0.02). Taken together, the overall QOL was significantly improved (Total QOL: OR = 13.95, CI = 8.27−19.63, *P* < 0.00001) (Figure 6).

### Immune function evaluation

The immune status of patients was examined before and after treatment. After CIK/DC-CIK treatment, percentages of CD3^+^, CD3^−^CD56^+^, CD3^+^CD56^+^ and CD16^+^CD56^+^ cells and CD4^+^/CD8^+^ ratio were significantly increased (Table 2 and Supplementary Figure 2, CD3^+^: OR = 5.46, CI = 3.18−7.74, *P* < 0.00001; CD3^−^CD56^+^: OR = 4.08, CI = 0.82−7.34, *P* = 0.01; CD3^+^CD56^+^: OR = 2.16, CI = 0.15−4.16, *P* = 0.03; CD4^+^/CD8^+^: OR = 0.39, CI = 0.24−0.53, *P* < 0.00001; CD16^+^CD56^+^: OR = 5.62, CI = 2.78−8.47, *P* = 0.0001), whereas proportions of CD4^+^, CD8^+^ and CD4^+^CD25^+^ were not significantly changed (CD4^+^: OR = 0.40, CI = −4.81−5.61, *P* = 0.88; CD8^+^: OR = 0.07, CI = −2.98−3.12, *P* = 0.97; CD4^+^CD25^+^: OR = −0.70, CI = −1.52–0.12, *P* = 0.10). Levels of IFN-γ and IL-2 were significantly increased after CIK/DC-CIK immunotherapy (Table 2 and Supplementary Figure 3, IFN-γ: OR = 5.28, CI = 2.21−8.36, *P* = 0.0008; IL-2: OR = 1.60, CI = 0.26−2.95, *P* = 0.02), while no obvious change was found in IL-6 and TNF-α expression (IL-6: OR = −1.30, CI = −2.85−0.26, *P* = 0.10; TNF-α: OR = 1.21, CI = −1.06−3.48, *P* = 0.30).

**Table 2:** Comparison of lymphocyte subsets and cytokines before CIK/DC-CIK treatment and after CIK/DC-CIK therapy

Parameter	CIK/DC-CIK after treatment (No.)	CIK/DC-CIK before treatment (No.)	Analysis method	Heterogeneity		Odds Ratio (OR)	95% CI	*P*-value
				*I*^2^ (%)	*P*-value			
CD3^+^	860	860	Random	94	< 0.00001	5.46	3.18 to 7.74	<0.00001
CD4^+^	860	860	Random	99	< 0.00001	0.40	−4.81 to 5.61	0.88
CD8^+^	808	808	Random	98	< 0.00001	0.07	−2.98 to 3.12	0.97
CD4^+^/CD8^+^	391	391	Random	87	< 0.00001	0.39	0.24 to 0.53	<0.00001
CD3^−^CD56^+^	441	441	Random	95	< 0.00001	4.08	0.82 to 7.34	0.01
CD3^+^CD56^+^	429	429	Random	99	< 0.00001	2.16	0.15 to 4.16	0.03
CD4^+^CD25^+^	393	393	Random	81	0.0001	−0.70	−1.52 to 0.12	0.10
CD16^+^CD56^+^	204	204	Random	97	< 0.00001	5.62	2.78 to 8.47	0.0001
IFN-γ	429	429	Random	99	< 0.00001	5.28	2.21 to 8.36	0.0008
IL-2	429	429	Random	92	< 0.00001	1.60	0.26 to 2.95	0.02
IL-6	92	92	Random	93	< 0.00001	−1.30	−2.85 to 0.26	0.10
TNF-α	337	337	Random	97	< 0.00001	1.21	−1.06 to 3.48	0.30

### Adverse events assessment

No serious adverse events or death occurrence was reported in patients receiving CIK/DC-CIK immunotherapy. Compared with patients in chemo-alone group, those in combined group suffered milder leucopenia, thrombocytopenia, nausea and vomiting, liver dysfunction, myelosuppression, peripheral neurotoxicity and gastrointestinal adverse reactions, except the effects on anemia [Table 3 and Supplementary Figures 4–9, leucopenia: OR = 0.51, CI = 0.32–0.82, *P* = 0.005 (leucopenia I: OR = 1.02, CI = 0.67–1.56, *P* = 0.92; leucopenia II: OR = 0.81, CI = 0.42–1.56, *P* = 0.53; leucopenia III: OR = 0.40, CI = 0.21–0.75, *P* = 0.004; leucopenia IV: OR = 0.55, CI = 0.26–1.19, *P* = 0.13); thrombocytopenia: OR = 0.54, CI = 0.29–0.99, *P* = 0.05 (thrombocytopenia I: OR = 0.69, CI = 0.42–1.13, *P* = 0.14; thrombocytopenia II: OR = 0.76, CI = 0.35–1.69, *P* = 0.50; thrombocytopenia III: OR = 0.24, CI = 0.10–0.54, *P* = 0.0006; thrombocytopenia IV: OR = 0.90, CI= 0.22–3.63, *P* = 0.88); nausea and vomiting: OR = 0.55, CI = 0.31–0.98, *P* = 0.04 (nausea and vomiting I: OR = 1.06, CI = 0.49–2.29, *P* = 0.88; nausea and vomiting II: OR = 0.61, CI = 0.42–0.89, *P* = 0.01; nausea and vomiting III: OR = 0.48, CI = 0.21–1.08, *P* = 0.08; nausea and vomiting IV: OR = 0.45, CI = 0.08–2.45, *P* = 0.35); liver dysfunction: OR = 0.55, CI = 0.38–0.80, *P* = 0.002 (liver dysfunction I: OR = 0.59, CI = 0.33–1.06, *P* = 0.08; liver dysfunction II: OR = 0.52, CI = 0.31–0.88, *P* = 0.01; liver dysfunction III: OR = 0.90, CI = 0.26–3.14, *P* = 0.87; liver dysfunction IV: OR = 0.53, CI = 0.13–2.26, *P* = 0.39); myelosuppression: OR = 0.21, CI = 0.11–0.39, *P* < 0.00001; peripheral neurotoxicity: OR = 0.31, CI = 0.18–0.55, *P* < 0.00001; gastrointestinal adverse reaction: OR = 0.26, CI = 0.15–0.45, *P* < 0.00001; anemia: OR = 0.52, CI = 0.26–1.05, *P* = 0.07 (anemia I: OR = 0.52, CI = 0.27–1.00, *P* = 0.05; anemia II: OR = 0.71, CI = 0.44–1.14, *P* = 0.15; anemia III: OR = 0.60, CI = 0.22–1.63, *P* = 0.32; anemia IV: OR = 0.45, CI = 0.13–1.51, *P* = 0.19)].

**Table 3:** Comparison of adverse events between the chemotherapy+CIK/DC-CIK and chemotherapy alone groups

Adverse events	Chemo+CIK/DC-CIK	Chemo	Analysis method	Heterogeneity		Odds Ratio (OR)	95% CI	*P*-value
	No. patients (*n*)	No. patients (*n*)		*I*^2^ (%)	*P*-value			
**Leucopenia**	412	384	Random	38	0.15	0.51	0.32 to 0.82	0.005
Leucopenia I	339	312	Random	0	0.48	1.02	0.67 to 1.56	0.92
Leucopenia II	339	312	Random	63	0.05	0.81	0.42 to 1.56	0.53
Leucopenia III	339	312	Random	23	0.28	0.40	0.21 to 0.75	0.004
Leucopenia IV	339	312	Random	0	1.00	0.55	0.26 to 1.19	0.13
**Anemia**	382	354	Random	75	0.003	0.52	0.26 to 1.05	0.07
Anemia I	339	312	Random	59	0.06	0.52	0.27 to 1.00	0.05
Anemia II	339	312	Random	0	0.55	0.71	0.44 to 1.14	0.15
Anemia III	339	312	Random	0	0.53	0.60	0.22 to 1.63	0.32
Anemia IV	339	312	Random	0	0.68	0.45	0.13 to 1.51	0.19
**Thrombocytopenia**	412	384	Random	63	0.02	0.54	0.29 to 0.99	0.05
Thrombocytopenia I	339	312	Random	23	0.27	0.69	0.42 to 1.13	0.14
Thrombocytopenia II	339	312	Random	55	0.08	0.76	0.35 to 1.69	0.50
Thrombocytopenia III	339	312	Random	0	0.96	0.24	0.10 to 0.54	0.0006
Thrombocytopenia IV	339	312	Random	0	0.88	0.90	0.22 to 3.63	0.88
**Nausea, vomiting**	427	399	Random	65	0.009	0.55	0.31 to 0.98	0.04
Nausea, vomiting I	339	312	Random	67	0.03	1.06	0.49 to 2.29	0.88
Nausea, vomiting II	339	312	Random	0	0.86	0.61	0.42 to 0.89	0.01
Nausea, vomiting III	339	312	Random	0	0.57	0.48	0.21 to 1.08	0.08
Nausea, vomiting IV	339	312	Random	0	1.00	0.45	0.08 to 2.45	0.35
**Liver dysfunction**	401	374	Random	0	0.44	0.55	0.38 to 0.80	0.002
Liver dysfunction I	298	272	Fixed	0	0.96	0.59	0.33 to 1.06	0.08
Liver dysfunction II	298	272	Fixed	0	0.57	0.52	0.31 to 0.88	0.01
Liver dysfunction III	298	272	Fixed	36	0.21	0.90	0.26 to 3.14	0.87
Liver dysfunction IV	298	272	Fixed	0	0.47	0.53	0.13 to 2.26	0.39
**Myelosuppression**	159	159	Random	0	0.90	0.21	0.11 to 0.39	< 0.00001
**Peripheral neurotoxicity**	196	195	Random	0	0.42	0.31	0.18 to 0.55	< 0.00001
**Gastrointestinal AE**	153	152	Random	0	0.53	0.26	0.15 to 0.45	< 0.00001

AE: adverse reaction.

### Sensitivity analysis

Firstly, a sensitivity analysis was conducted and 8 trials [18, 30, 32, 38, 44, 49, 52, 55] were excluded because Folfox/Xelox/Folfiri regimen was not applied to all patients. The results of this analysis were similar to those obtained from the overall analysis of the pooled trials.

Secondly, CRC patients were treated by DC-CIK immunotherapy in 20 trials and CIK alone in the other 9 trials. Studies were grouped according to different immunotherapy strategies (CIK or DC-CIK), and pooled results were compared. The comparison showed both CIK and DC-CIK were effective in treating CRC, and no obvious difference was observed in most pooled analyses. (Tables 4 and 5, Supplementary Figures 10–13).

**Table 4:** Meta-analysis of OS, DFS, ORR and DCR in Chemo-DC-CIK and Chemo-CIK subgroups

Immunotherapy type (subgroup)	Parameters	Chemo+CIK/DC-CIK	Chemo	Analysis method	Heterogeneity		Odds Ratio (OR)	95% CI	*P*-value
		No. patients (*n*)	No. patients (*n*)		*I*^2^ (%)	*P*-value			
DC-CIK	1-year OS	273	313	Fixed	0	0.94	2.02	1.05 to 3.89	0.04
	2-year OS	270	317	Fixed	0	0.90	2.43	1.55 to 3.83	0.0001
	3-year OS	151	192	Fixed	26	0.25	2.73	1.64 to 4.55	0.0001
	1-year DFS	106	147	Fixed	0	0.60	4.54	2.26 to 9.11	< 0.0001
	2-year DFS	92	133	Fixed	0	0.98	2.45	1.40 to 4.28	0.002
	3-year DFS	106	147	Fixed	50	0.13	2.41	1.43 to 4.08	0.001
	ORR	547	539	Fixed	0	0.99	1.85	1.44 to 2.37	< 0.00001
	DCR	547	539	Fixed	0	0.94	2.94	2.20 to 3.93	< 0.00001
CIK	1-year OS	290	280	Fixed	20	0.29	2.36	1.44 to 3.86	0.0006
	2-year OS	209	200	Fixed	35	0.21	2.78	1.70 to 4.54	< 0.0001
	3-year OS	206	198	Fixed	0	0.83	2.37	1.53 to 3.65	0.0001
	1-year DFS	105	97	Fixed	0	0.35	3.17	1.59 to 6.33	0.001
	2-year DFS	105	97	Fixed	0	0.63	2.51	1.41 to 4.46	0.002
	3-year DFS	105	97	Fixed	0	1.00	1.98	1.11 to 3.52	0.02
	ORR	223	218	Fixed	0	0.53	1.79	1.16 to 2.78	0.009
	DCR	223	218	Fixed	0	0.64	2.33	1.37 to 3.97	0.002

**Table 5:** Meta-analysis of immunophenotype in Chemo-DC-CIK and Chemo-CIK subgroups

Immunotherapy type (subgroup)	Parameters	CIK/DC-CIK after treatment (No.)	CIK/DC-CIK Before treatment (No.)	Analysis method	Heterogeneity		Odds Ratio (OR)	95% CI	*P*-value
					*I*^2^ (%)	*P*-value			
DC-CIK	CD3^+^	661	661	Random	80	< 0.00001	4.33	2.15 to 6.50	< 0.0001
	CD4^+^	661	661	Random	99	< 0.00001	−0.48	−8.03 to 7.07	0.90
	CD8^+^	609	609	Random	93	< 0.00001	0.27	−2.08 to 2.61	0.82
	CD4^+^/CD8^+^	252	252	Random	91	< 0.00001	0.34	0.19 to 0.50	< 0.0001
	CD3^+^CD56^+^	369	369	Random	99	< 0.00001	1.16	−1.05 to 3.38	0.30
	CD16^+^CD56^+^	108	108	Random	55	0.11	6.95	5.67 to 8.23	<0.00001
CIK	CD3^+^	199	199	Random	97	< 0.00001	7.85	3.54 to 12.17	0.0004
	CD4^+^	199	199	Random	95	< 0.00001	3.62	0.10 to 7.13	0.04
	CD8^+^	199	199	Random	99	< 0.00001	−0.47	−6.82 to 5.87	0.88
	CD4^+^/CD8^+^	139	139	Random	3	0.36	0.70	0.36 to 1.03	< 0.0001
	CD3^+^CD56^+^	60	60	Random	0	0.36	4.66	3.86 to 5.46	< 0.00001
	CD16^+^CD56^+^	96	96	Random	99	< 0.00001	3.26	−2.72 to 9.23	0.29

### Publication bias

Funnel plots drawn for the studies on the OS, ORR, DCR and lymphocyte subsets percentages of CD3^+^, CD4^+^, CD8^+^ and CD4^+^/CD8^+^ were symmetrical in general, indicating passably controlled publication bias (Supplementary Figure 14). In addition, publication bias was further analyzed by both Begg's and Egger's regression asymmetry tests (Table 6). Bias was observed in 3-year OS (Egger: 0.016; Begg: 0.386) and percentages of CD8^+^ subsets (Egger: 0.008; Begg: 0.284), but not in other pooled-analyses. We further evaluated whether the observed publication bias significantly influenced the pooled risk using trim and filled method. The adjusted OR indicated same trend with the result of the primary analysis (3-year OS: before: *P* < 0.00001, after: *P* < 0.0001; CD8^+^: before: *P* = 0.966, after: *P* = 0.966). This publication bias analysis confirmed the reliability of our primary conclusions, except those based on few numbers of trials.

**Table 6:** Publication bias on OS, ORR, DCR and lymphocyte subsets (CD3^+^, CD4^+^, CD8^+^ and CD4^+^/CD8^+^)

Publication Bias	1-year OS	2-year OS	3-year OS	ORR	DCR	CD3	CD4	CD8	CD4/CD8
**Begg**	0.100	0.640	0.386	0.289	0.256	0.880	0.028	0.284	0.474
**Egger**	0.630	0.390	0.016	0.198	0.195	0.554	0.282	0.008	0.679

Parameters discussed in over 8 papers were conducted bias analyses.

## DISCUSSION

In recent years, there have been several clinical trials using CIK/DC-CIK immunotherapy to treat CRC [5, 18]. However, the exact therapeutic effects remain unclear because of sample sizes variability and unstandardized clinical trial protocols. To address this problem, we performed an extensive online search followed by rigorous data analysis. We studied 29 clinical trials including 1300 CRC patients who received CIK/DC-CIK immunotherapy. Our meta-analysis revealed that the combination of CIK/DC-CIK immunotherapy and chemotherapy for CRC could improve OS, ORR, DCR, patients’ life quality and immune function, and alleviates the adverse events caused by chemotherapy.

This meta-analysis confirmed the safety of CIK/DC-CIK immunotherapy for CRC, and its side effects were tolerated by all patients without causing serious adverse events or death after therapy. The adverse events caused by chemotherapy, including leucopenia, thrombocytopenia, nausea and vomiting, liver dysfunction, myelosuppression, peripheral neurotoxicity and gastrointestinal adverse reaction were obviously alleviated by CIK/DC-CIK immunotherapy (*P* < 0.05). The combination therapy also improved the quality of life of patients reflected by enhanced physical, role, emotion, cognize and society function (*P* < 0.05). Furthermore, CIK/DC-CIK immunotherapy also enhanced the efficiency of chemotherapy for CRC. Compare to patients treated by chemotherapy alone, patients with combined therapy showed markedly increased OS, DFS, ORR and DCR.

The immunosuppressed status of cancer patients has been reported previously [3]. Therefore, immune system reconstruction is one of the key factors to effectively treat malignant tumors. Our analysis showed significantly increased percentages of CD3^+^, CD4^+^/CD8^+^, CD3^−^CD56^+^, CD3^+^CD56^+^ and CD16^+^CD56^+^ T cells upon CIK/DC-CIK treatment, indicating that immune function of CRC patients was improved after CIK/DC-CIK immunotherapy [56, 57]. However, no significant difference was shown in percentages of CD4*+*, CD8*+* and CD4^+^CD25^+^ T cells between pre and post immunotherapy. This may be related to the different time points of T-lymphocyte subset were tested in these trials [19] and the various CIK/DC-CIK transfusion dosages may also cause different immune responses. Th1 cytokines, including IFN-γ, IL-2, and TNF-α, enhance the cytotoxicity of CIK cells, whereas Th2 cytokines like IL-6 and IL-10 is associated with tumor immune escape [3, 58]. The balance between Th1 and Th2 cells is crucial in immunotherapy [3, 4]. Our analysis showed that after CIK/DC-CIK immunotherapy for CRC patients, IFN-γ and IL-2 levels were significantly increased, whereas no significant differences were observed in TNF-α and IL-6, indicating a more important role of Th1 than Th2 cytokines.

The determination of optimal therapeutic strategy is valuable for CRC treatment. To optimized the therapeutic protocol, there are several aspects need to be considered. First of all, the phenotype of *in vitro* cultured CIK cells is associated with treatment outcomes of immunotherapy. Low percentages of CD3^+^CD4^+^ subset and high percentages of CD3^+^CD8^+^ and CD3^+^CD56^+^ subset were reported associated with improved OS in CRC patients [56]. Therefore, the immune phenotype of CIK cells could be an important criterion for CIK/DC-CIK immunotherapy. However, relevant studies were insufficient and need more research evidence to support this conclusion. Secondly, to determine the usage of CIK or DC-CIK, the difference between their therapeutic effects should be evaluated. In our analysis, CIK and DC-CIK subgroup analyses showed that both were effective in treating CRC. *In vitro* experiments showed that DC-CIK had a better antitumor effect than CIK alone, but no obvious difference was observed in most pooled analysis in treating CRC patients. Moreover, the choose of chemotherapy regimen is also important for the determination of optimal therapeutic strategy. Folfox, Xelox and Folfiri are the three most common chemotherapy regimens with similar therapeutic effects and have been considered as first-line treatments for CRC [59–61]. Sensitivity analysis was conducted in this research and trials in which Folfox, Xelox, or Folfiri were not applied to all patients were excluded. The results of this analysis indicated that Folfox/Xelox/Folfiri combined with CIK/DC-CIK immunotherapy is effective for treating CRC. To summarize, we expect that our study will be valuable for the design of more comprehensive clinical trials in the future.

There are some limitations in our study. Firstly, the investigated 29 trials were conducted in the Chinese population, and this analysis did not go through the open external evaluation, which may lead to an overestimation of treatment effects. Furthermore, insufficient information of some patients, the small total sample sizes and other variables may introduce bias into conclusions. Moreover, the therapeutic effects of CIK/DC-CIK immunotherapy are affected by numerous factors such as injection modes, tumor stage and transfer cycles [5, 17, 30, 45]. Further detailed analyses need to be conducted based on papers with complete information, standardized therapeutic regimens and restrict patients involving criterion.

Taken together, this research demonstrated that the combination of CIK/DC-CIK immunotherapy and chemotherapy was safe and applicable for CRC patients. It markedly prolongs survival time, enhances immune function, and improves the efficacy of the treatment of CRC patients. Therefore, CIK/DC-CIK immunotherapy is an effective therapy for CRC treatment.

## MATERIALS AND METHODS

### Search strategy and selection criteria

Literatures were searched across Cochrane Library, EMBASE, PubMed, Wanfang and CNKI databases with key terms “dendritic cells”, “immunotherapy”, “cytokine-induced killer cells” or “DC-CIK” combined with “colorectal cancer”. No language limits were applied. The initial search was performed in February 2016 and updated in September 2016.

Trials meet the following criteria were selected into this analysis: (1) Studies were concerned with advanced CRC. (2) Patients in experimental group underwent chemotherapy combined with CIK /DC-CIK immunotherapy, and patients in control group were treated by chemotherapy alone.

### Data collection and quality assessment

Data were extracted from all selected literatures independently by two investigators, and discrepancy was resolved by discussing with a third investigator. Collected information includes: name of first author; years of publication; numbers of subjects; patient ages; tumor stages; experiment regimens; chemotherapy regimens; *in vitro* cell culture conditions, and utilized immune cells numbers.

The quality of the included articles was evaluated according to Cochrane Handbook [62].

### Curative effects

Clinical responses include prognosis and treatment efficacy. Prognosis was estimated by OS and DFS. Treatment efficacy was assessed in terms of the CR, PR, SD, PD, ORR (ORR = CR+PR), DCR (DCR = CR+PR+SD) and patients’ QOL. OS was defined as the length of time from the start of treatment to death from any cause [63]. DFS was defined as length of time from the start of treatment to the first recurrence evidence or death [3]. The immune function of CRC patients before and after treatment was determined by lymphocyte subsets percentages (CD3^+^, CD4^+^, CD8^+^, CD3^−^CD56^+^, CD3^+^CD56^+^, CD16^+^CD56^+^ and CD4^+^CD25^+^) and cytokines secretion levels (IFN-γ, IL-2, IL-4, IL-6 and TNF-α). Adverse events in included trials were also taken into assessment.

### Statistical analysis

Review Manager (version 5.2, Nordic Cochran Centre, Copenhagen, Denmark) and Stata Statistical Software (version 12.0, Stata Corp., College Station, TX, USA) was used for analyses. *P* < 0.05 was considered statistically significant. Heterogeneity among the involved trials was assessed to determine the most suitable model [64]. A random-effects method would be applied when heterogeneity existed; otherwise, a fixed-effects method would be used. Cochran's Q test was performed to determine the homogeneity of our results; homogeneity was considered at *I^2^* < 50% or *P* > 0.1. Odds ratios (OR) were the principal measures of effect and were presented with a 95% confidence interval (CI).

Publication bias was analyzed by Begg's and Egger's regression asymmetry tests [65], and visually evaluated using the funnel plot. When publication bias existed, trim-and-fill method was applied to adjust the pooled estimates of potentially unpublished studies. Corrected results were compared with the original pooled OR. Sensitivity analysis was conducted and 8 trials [18, 30, 32, 38, 44, 49, 52, 55] were excluded because Folfox/Xelox/Folfiri regimen was not applied to all patients.

## SUPPLEMENTARY MATERIALS FIGURES AND TABLES




