# Supplementary material for: Cytokine-induced killer cells/dendritic cells-cytokine induced killer cells immunotherapy combined with chemotherapy for treatment of colorectal cancer in China: a meta-analysis of 29 trials involving 2,610 patients
Source: Oncotarget. 2017 Mar 29;8(28):45164–77. doi: 10.18632/oncotarget.16665 (PMC5542175; doi:10.18632/oncotarget.16665)
Supplement: Supplementary file 1 [file oncotarget-08-45164-s001.pdf]

Cytokine-induced killer cells/dendritic cells-cytokine induced killer cells immunotherapy combined with chemotherapy for treatment of colorectal cancer in China: a meta-analysis of 29 trials involving 2,610 patients

Supplementary Materials

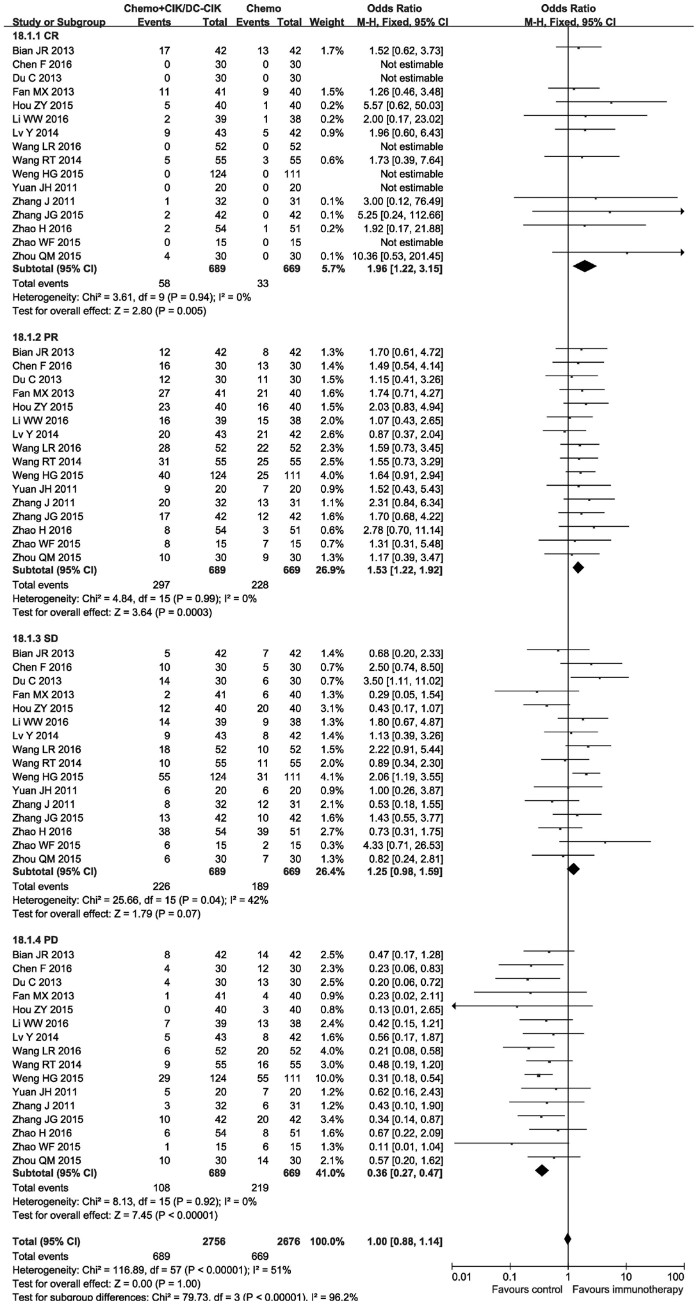

Supplementary Figure 1: Forest plot of the comparison of complete response rates (CR), partial response rates (PR), stable disease rates (SD) and progressive disease rates (PD). CI, confidence interval; Chemo, chemotherapy; CIK/DC-CIK, CIK/DC- CIK immunotherapy. The fixed-effects meta-analysis model (Mantel–Haenszel method) was used.

Study or Subgroup	Post-CIK/DC-CIK Mean	SD	Total	Pre-CIK/DC-CIK Mean	SD	Total	Weight	Mean Difference IV, Random, 95% CI
**7.1.1 CD3+**								
Bian JR 2013	75.3	15.8	42	71.3	14.2	42	0.3%	4.00 [-2.42, 10.42]
Chen F 2016	65.7	10.6	30	61.1	14.1	30	0.4%	4.60 [-1.71, 10.91]
Du C 2013	77.5	5.6	30	66.8	5.4	30	1.0%	10.70 [7.92, 13.48]
Fan MX 2013	67.1	1.2	41	62.5	2.2	41	1.6%	4.60 [3.83, 5.37]
Li S 2012	59.6	3.8	20	60.4	3	20	1.2%	-0.80 [-2.92, 1.32]
Li WW 2016	76.2	11.3	39	68.2	11.4	39	0.5%	8.00 [2.96, 13.04]
Lin T 2015	68.7	13.9	134	71.4	14.7	134	0.8%	-2.70 [-6.13, 0.73]
Lv Y 2014	74.5	2.3	43	62.3	2.3	43	1.5%	12.20 [11.23, 13.17]
Rui T 2015	61.9	3.8	45	60.3	3.9	45	1.3%	1.60 [0.01, 3.19]
Wang LR 2016	61.5	9.3	52	56.4	8.1	52	0.8%	5.10 [1.75, 8.45]
Wang R 2016	74.4	5.6	42	67.7	5.3	42	1.1%	6.70 [4.37, 9.03]
Wang RT 2014	73.9	8.1	55	67.2	5.1	55	1.0%	6.70 [4.17, 9.23]
Weng HG 2015	76.2	14.1	124	72.9	14.7	124	0.8%	3.30 [-0.29, 6.89]
Weng JF 2014	78.4	11.5	41	67.7	9.8	41	0.6%	10.70 [6.08, 15.32]
Yin LW 2013	74.6	15.6	40	71.2	14.3	40	0.3%	3.40 [-3.16, 9.96]
Yuan JH 2011	64	7	20	54	10	20	0.5%	10.00 [4.65, 15.35]
Zhang J 2011	58	7.9	32	52	7.1	32	0.7%	6.00 [2.32, 9.68]
Zhang JY 2014	69.7	13.9	30	65.6	10	30	0.4%	4.10 [-2.03, 10.23]
**Subtotal (95% CI)**			**860**			**860**	**14.6%**	**5.46 [3.18, 7.74]**
Heterogeneity: Tau² = 20.42; Chi² = 284.09, df = 17 (P < 0.00001); I² = 94%								
Test for overall effect: Z = 4.69 (P < 0.00001)								
**7.1.2 CD4+**								
Bian JR 2013	36.8	6.9	42	37.7	7.8	42	0.9%	-0.90 [-4.05, 2.25]
Chen F 2016	31.6	10.8	30	39.4	9.5	30	0.5%	-7.80 [-12.95, -2.65]
Du C 2013	46.7	1.7	30	37.9	3.5	30	1.4%	8.80 [7.41, 10.19]
Fan MX 2013	37.8	4.3	41	31.5	3.5	41	1.3%	6.30 [4.60, 8.00]
Li S 2012	37.1	5.7	20	30.2	3.9	20	0.9%	6.90 [3.87, 9.93]
Li WW 2016	36	9.6	39	37.9	12.3	39	0.5%	-1.90 [-6.80, 3.00]
Lin T 2015	3.5	6.6	134	36.7	8.2	134	1.3%	-33.20 [-34.98, -31.42]
Lv Y 2014	42.3	2.4	43	33.6	1.3	43	1.5%	8.70 [7.88, 9.52]
Rui T 2015	34.1	3.7	45	30.8	2.6	45	1.4%	3.30 [1.98, 4.62]
Wang LR 2016	38.1	8.4	52	32.4	8.6	52	0.8%	5.70 [2.43, 8.97]
Wang R 2016	45.9	3.6	42	40.2	3.7	42	1.3%	5.70 [4.14, 7.26]
Wang RT 2014	56.5	8.6	55	55.1	9.2	55	0.8%	1.40 [-1.93, 4.73]
Weng HG 2015	35.9	6.6	124	37	8.2	124	1.2%	-1.10 [-2.95, 0.75]
Weng JF 2014	40.5	6.6	41	30.6	5.5	41	1.0%	9.90 [7.27, 12.53]
Yin LW 2013	34.7	5.6	40	37.4	7.3	40	0.9%	-2.70 [-5.55, 0.15]
Yuan JH 2011	34	8	20	30	6	20	0.6%	4.00 [-0.38, 8.38]
Zhang J 2011	36	8.7	32	30	8.1	32	0.6%	6.00 [1.88, 10.12]
Zhang JY 2014	25.4	10.9	30	38	9.9	30	0.5%	-12.60 [-17.87, -7.33]
**Subtotal (95% CI)**			**860**			**860**	**17.6%**	**0.40 [-4.81, 5.61]**
Heterogeneity: Tau² = 124.58; Chi² = 1997.67, df = 17 (P < 0.00001); I² = 99%								
Test for overall effect: Z = 0.15 (P = 0.88)								
**7.1.3 CD8+**								
Bian JR 2013	30.7	7.3	42	27.3	7	42	0.9%	3.40 [0.34, 6.46]
Chen F 2016	39.1	8.1	30	31.9	7.6	30	0.7%	7.20 [3.23, 11.17]
Du C 2013	30.8	3.3	30	29.8	2	30	1.4%	1.00 [-0.38, 2.38]
Fan MX 2013	23.7	3	41	30.8	3.1	41	1.4%	-7.10 [-8.42, -5.78]
Li S 2012	28.8	4.9	20	32.1	3.8	20	1.0%	-3.30 [-6.02, -0.58]
Li WW 2016	38	8.5	39	28.6	4.6	39	0.9%	9.40 [6.37, 12.43]
Lin T 2015	25.8	6.5	134	27.4	7.1	134	1.3%	-1.60 [-3.23, 0.03]
Lv Y 2014	22.3	1.7	43	33.3	2.2	43	1.5%	-11.00 [-11.83, -10.17]
Rui T 2015	26	4.4	45	30.8	4	45	1.3%	-4.80 [-6.54, -3.06]
Wang R 2016	29.1	2.7	42	27.4	2.9	42	1.5%	1.70 [0.50, 2.90]
Wang RT 2014	44	2.4	55	43.7	12.4	55	0.8%	0.30 [-3.04, 3.64]
Weng HG 2015	30	6.5	124	28.4	7.1	124	1.3%	1.60 [-0.09, 3.29]
Weng JF 2014	26.6	5.2	41	34.7	7	41	1.0%	-8.10 [-10.77, -5.43]
Yin LW 2013	30.2	6.3	40	27.2	6.1	40	1.0%	3.00 [0.28, 5.72]
Yuan JH 2011	27	4	20	29	5	20	1.0%	-2.00 [-4.81, 0.81]
Zhang J 2011	28	6.2	32	30	6.8	32	0.8%	-2.00 [-5.19, 1.19]
Zhang JY 2014	35.8	11.6	30	19.7	6.3	30	0.5%	16.10 [11.38, 20.82]
**Subtotal (95% CI)**			**808**			**808**	**18.3%**	**0.07 [-2.98, 3.12]**
Heterogeneity: Tau² = 39.32; Chi² = 720.07, df = 16 (P < 0.00001); I² = 98%								
Test for overall effect: Z = 0.04 (P = 0.97)								
**7.1.4 CD4+/CD8+**								
Fan MX 2013	1.6	1.4	41	1	1.1	41	1.6%	0.60 [0.06, 1.14]
Li S 2012	1.6	0.3	20	1	0.3	20	1.6%	0.60 [0.41, 0.79]
Lv Y 2014	1.9	1.4	43	1	0.6	43	1.6%	0.90 [0.44, 1.36]
Rui T 2015	1.3	0.3	45	1	0.2	45	1.6%	0.30 [0.19, 0.41]
Wang LR 2016	1.3	0.2	52	1.1	0.2	52	1.6%	0.20 [0.12, 0.28]
Wang R 2016	1.6	0.2	42	1.5	0.5	42	1.6%	0.10 [-0.06, 0.26]
Wang RT 2014	2.3	2.4	55	2.1	2.4	55	1.5%	0.20 [-0.70, 1.10]
Weng JF 2014	1.5	0.2	41	0.9	0.2	41	1.6%	0.60 [0.51, 0.69]
Yuan JH 2011	1.9	0.6	20	1.6	0.7	20	1.6%	0.30 [-0.10, 0.70]
Zhang J 2011	1.3	0.2	32	1	0.3	32	1.6%	0.30 [0.18, 0.42]
**Subtotal (95% CI)**			**391**			**391**	**16.2%**	**0.39 [0.24, 0.53]**
Heterogeneity: Tau² = 0.04; Chi² = 70.71, df = 9 (P < 0.00001); I² = 87%								
Test for overall effect: Z = 5.19 (P < 0.00001)								
**7.1.5 CD3-CD56+**								
Bian JR 2013	14.3	5.9	42	11.3	4	42	1.2%	3.00 [0.84, 5.16]
Chen F 2016	16.5	7.5	30	14.7	5.2	30	0.8%	1.80 [-1.47, 5.07]
Lin T 2015	11.1	6.6	134	12.4	4.4	134	1.4%	-1.30 [-2.64, 0.04]
Weng HG 2015	12.6	6.1	124	11.4	4.4	124	1.4%	1.20 [-0.12, 2.52]
Weng JF 2014	17.6	3.8	41	9.7	2.9	41	1.4%	7.90 [6.44, 9.36]
Yin LW 2013	14.6	6.3	40	11.2	4.3	40	1.1%	3.40 [1.04, 5.76]
Zhang JY 2014	14.4	9.5	30	1	1.2	30	0.8%	13.40 [9.97, 16.83]
**Subtotal (95% CI)**			**441**			**441**	**8.1%**	**4.08 [0.82, 7.34]**
Heterogeneity: Tau² = 17.99; Chi² = 125.44, df = 6 (P < 0.00001); I² = 95%								
Test for overall effect: Z = 2.45 (P = 0.01)								
**7.1.6 CD3+CD56+**								
Chen F 2016	8.7	4.9	30	4.9	3.7	30	1.1%	3.80 [1.60, 6.00]
Du C 2013	10.1	1.6	30	5.5	1.6	30	1.5%	4.60 [3.79, 5.41]
Lin T 2015	3.1	1.7	134	4.8	1	134	1.6%	-1.70 [-2.03, -1.37]
Weng HG 2015	4.5	1.9	124	4.8	2.1	124	1.6%	-0.30 [-0.80, 0.20]
Weng JF 2014	3.6	0.8	41	1.1	0.2	41	1.6%	2.50 [2.25, 2.75]
Yin LW 2013	5.9	3.6	40	3.9	2.3	40	1.4%	2.00 [0.68, 3.32]
Zhang JY 2014	20.9	12.5	30	13.8	7.9	30	0.5%	7.10 [1.81, 12.39]
**Subtotal (95% CI)**			**429**			**429**	**9.4%**	**2.16 [0.15, 4.16]**
Heterogeneity: Tau² = 6.50; Chi² = 502.27, df = 6 (P < 0.00001); I² = 99%								
Test for overall effect: Z = 2.11 (P = 0.03)								
**7.1.7 CD4+CD25+**								
Chen F 2016	6.1	2.4	30	6.9	2.1	30	1.5%	-0.80 [-1.94, 0.34]
Li S 2012	4.2	2.2	20	6.6	1.8	20	1.4%	-2.40 [-3.65, -1.15]
Lin T 2015	6.5	1.5	134	7.9	1.3	134	1.6%	-1.40 [-1.74, -1.06]
Rui T 2015	11.8	4.7	45	9.4	4.4	45	1.2%	2.40 [0.52, 4.28]
Weng HG 2015	7.4	2.5	124	7.9	3.3	124	1.6%	-0.50 [-1.23, 0.23]
Yin LW 2013	6.9	2.3	40	7.2	2.4	40	1.5%	-0.30 [-1.33, 0.73]
**Subtotal (95% CI)**			**393**			**393**	**8.8%**	**-0.70 [-1.52, 0.12]**
Heterogeneity: Tau² = 0.76; Chi² = 25.69, df = 5 (P = 0.0001); I² = 81%								
Test for overall effect: Z = 1.67 (P = 0.10)								
**7.1.8 CD16+CD56+**								
Fan MX 2013	18.4	0.8	41	12.1	2.3	41	1.6%	6.30 [5.55, 7.05]
Li S 2012	19.3	5	20	10	3	20	1.0%	9.30 [6.74, 11.86]
Lv Y 2014	20.3	2.2	43	13.7	1.3	43	1.6%	6.60 [5.84, 7.36]
Rui T 2015	20.4	4.6	45	14.2	2.6	45	1.3%	6.20 [4.66, 7.74]
Wang RT 2014	2.3	2.4	55	2.1	2.4	55	1.5%	0.20 [-0.70, 1.10]
**Subtotal (95% CI)**			**204**			**204**	**7.0%**	**5.62 [2.78, 8.47]**
Heterogeneity: Tau² = 10.00; Chi² = 151.68, df = 4 (P < 0.00001); I² = 97%								
Test for overall effect: Z = 3.87 (P = 0.0001)								
**Total (95% CI)**			**4386**			**4386**	**100.0%**	**1.69 [1.27, 2.12]**
Heterogeneity: Tau² = 2.84; Chi² = 5577.22, df = 87 (P < 0.00001); I² = 98%								
Test for overall effect: Z = 7.84 (P < 0.00001)								
Test for subgroup differences: Chi² = 46.61, df = 7 (P < 0.00001), I² = 85.0%								

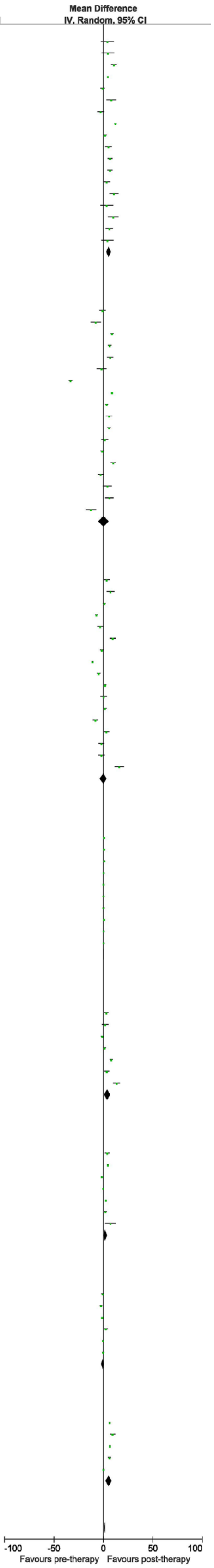

Supplementary Figure 2: Forest plot of the comparison of immunophenotype in pre- and post-CIK/DC-CIK immunotherapy. CI, confidence interval; CIK/DC-CIK, CIK/DC-CIK immunotherapy. The random effects meta-analysis model (Mantel–Haenszel method) was used.

Study or Subgroup	Post-CIK/DC-CIK Mean	Post-CIK/DC-CIK SD	Post-CIK/DC-CIK Total	Pre-CIK/DC-CIK Mean	Pre-CIK/DC-CIK SD	Pre-CIK/DC-CIK Total	Weight	Mean Difference IV, Random, 95% CI
**8.1.1 IFN-γ**								
Chen F 2016	32.1	2.4	30	22.2	2.6	30	4.7%	9.90 [8.63, 11.17]
Li S 2012	31.5	2.2	20	22.5	2.5	20	4.6%	9.00 [7.54, 10.46]
Li WW 2016	13.6	1.8	39	8.1	2	39	4.8%	5.50 [4.66, 6.34]
Lin T 2015	7.7	3.4	134	8.3	2.3	134	4.9%	-0.60 [-1.30, 0.10]
Wang R 2016	10.6	2.3	42	7.6	1.3	42	4.8%	3.00 [2.20, 3.80]
Weng HG 2015	8.8	4.1	124	8.1	2.5	124	4.8%	0.70 [-0.15, 1.55]
Yin LW 2013	16.9	3.4	40	7.2	2.3	40	4.7%	9.70 [8.43, 10.97]
**Subtotal (95% CI)**			**429**			**429**	**33.2%**	**5.28 [2.21, 8.36]**
Heterogeneity: Tau² = 16.92; Chi² = 442.84, df = 6 (P < 0.00001); I² = 99%								
Test for overall effect: Z = 3.37 (P = 0.0008)								
**8.1.2 IL-2**								
Chen F 2016	13.2	1.6	30	9.1	3.3	30	4.6%	4.10 [2.79, 5.41]
Li S 2012	11.4	1.5	20	10.3	1.9	20	4.7%	1.10 [0.04, 2.16]
Li WW 2016	10.4	2.8	39	7.4	2	39	4.7%	3.00 [1.92, 4.08]
Lin T 2015	10	3	134	10.7	4	134	4.8%	-0.70 [-1.55, 0.15]
Wang R 2016	11.2	2.2	42	8.1	1.4	42	4.8%	3.10 [2.31, 3.89]
Weng HG 2015	11.1	3	124	10.8	4.2	124	4.8%	0.30 [-0.61, 1.21]
Yin LW 2013	10.5	2.9	40	10.1	3.6	40	4.6%	0.40 [-1.03, 1.83]
**Subtotal (95% CI)**			**429**			**429**	**33.2%**	**1.60 [0.26, 2.95]**
Heterogeneity: Tau² = 3.00; Chi² = 73.76, df = 6 (P < 0.00001); I² = 92%								
Test for overall effect: Z = 2.33 (P = 0.02)								
**8.1.3 IL-6**								
Chen F 2016	4.4	2.3	30	6.9	3.2	30	4.6%	-2.50 [-3.91, -1.09]
Li S 2012	5.3	1.1	20	7	1.4	20	4.8%	-1.70 [-2.48, -0.92]
Wang R 2016	2.7	0.5	42	2.7	0.5	42	4.9%	0.00 [-0.21, 0.21]
**Subtotal (95% CI)**			**92**			**92**	**14.4%**	**-1.30 [-2.85, 0.26]**
Heterogeneity: Tau² = 1.68; Chi² = 27.68, df = 2 (P < 0.00001); I² = 93%								
Test for overall effect: Z = 1.63 (P = 0.10)								
**8.1.4 TNF-a**								
Li WW 2016	13.3	1.8	39	8.3	2.6	39	4.8%	5.00 [4.01, 5.99]
Lin T 2015	7.6	2.5	134	8.3	3.4	134	4.8%	-0.70 [-1.41, 0.01]
Weng HG 2015	8.7	2.1	124	8.4	3.3	124	4.9%	0.30 [-0.39, 0.99]
Yin LW 2013	8.2	1.3	40	7.9	3.1	40	4.8%	0.30 [-0.74, 1.34]
**Subtotal (95% CI)**			**337**			**337**	**19.2%**	**1.21 [-1.06, 3.48]**
Heterogeneity: Tau² = 5.17; Chi² = 88.61, df = 3 (P < 0.00001); I² = 97%								
Test for overall effect: Z = 1.04 (P = 0.30)								
**Total (95% CI)**			**1287**			**1287**	**100.0%**	**2.31 [1.14, 3.48]**
Heterogeneity: Tau² = 7.22; Chi² = 960.07, df = 20 (P < 0.00001); I² = 98%								
Test for overall effect: Z = 3.87 (P = 0.0001)								
Test for subgroup differences: Chi² = 16.60, df = 3 (P = 0.0009), I² = 81.9%								

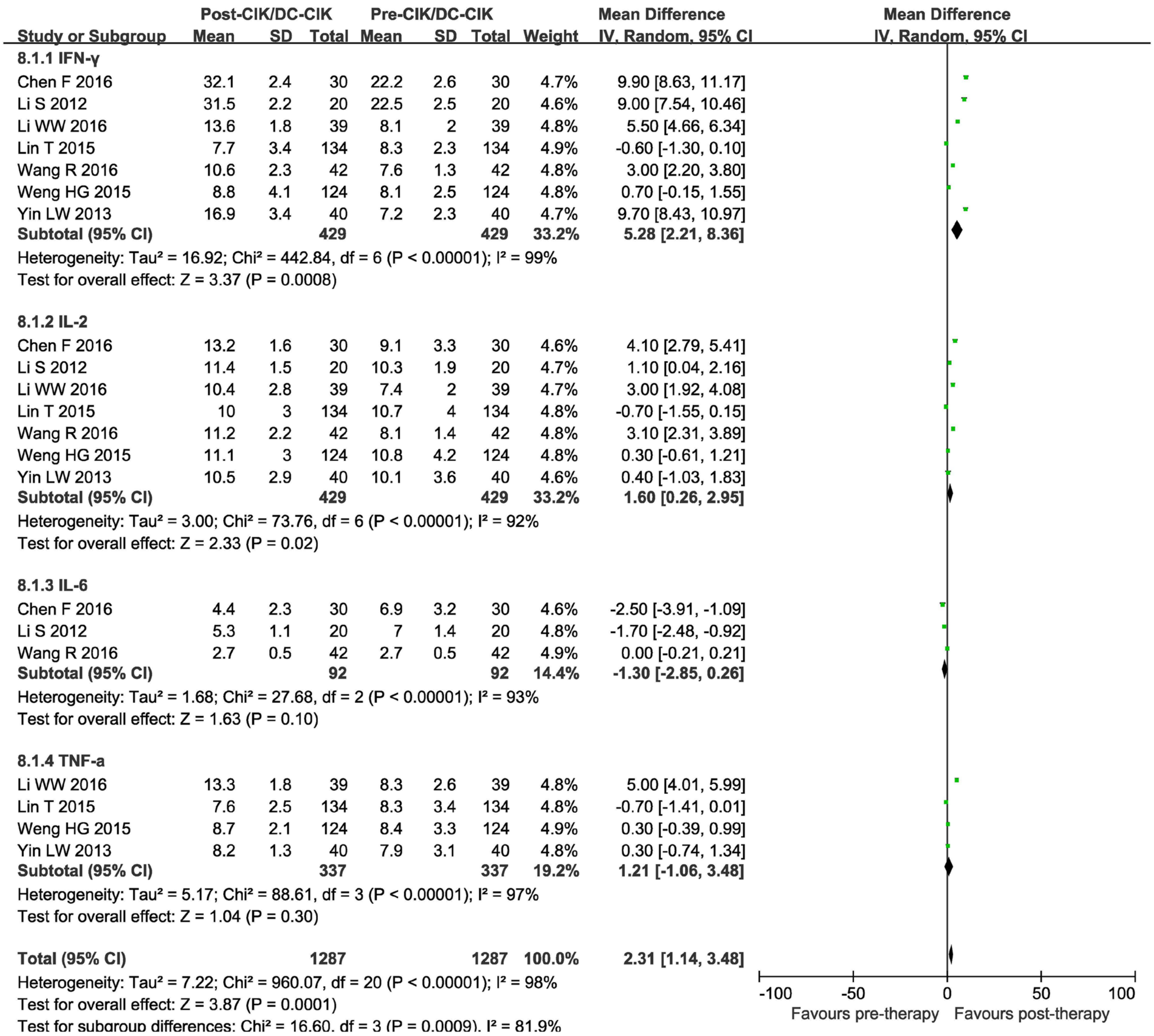

Supplementary Figure 3: Forest plot of the comparison of cytokines in pre- and post-CIK/DC-CIK immunotherapy. CI, confidence interval; CIK/DC-CIK, CIK/DC- CIK immunotherapy. The random effects meta-analysis model (Mantel–Haenszel method) was used.

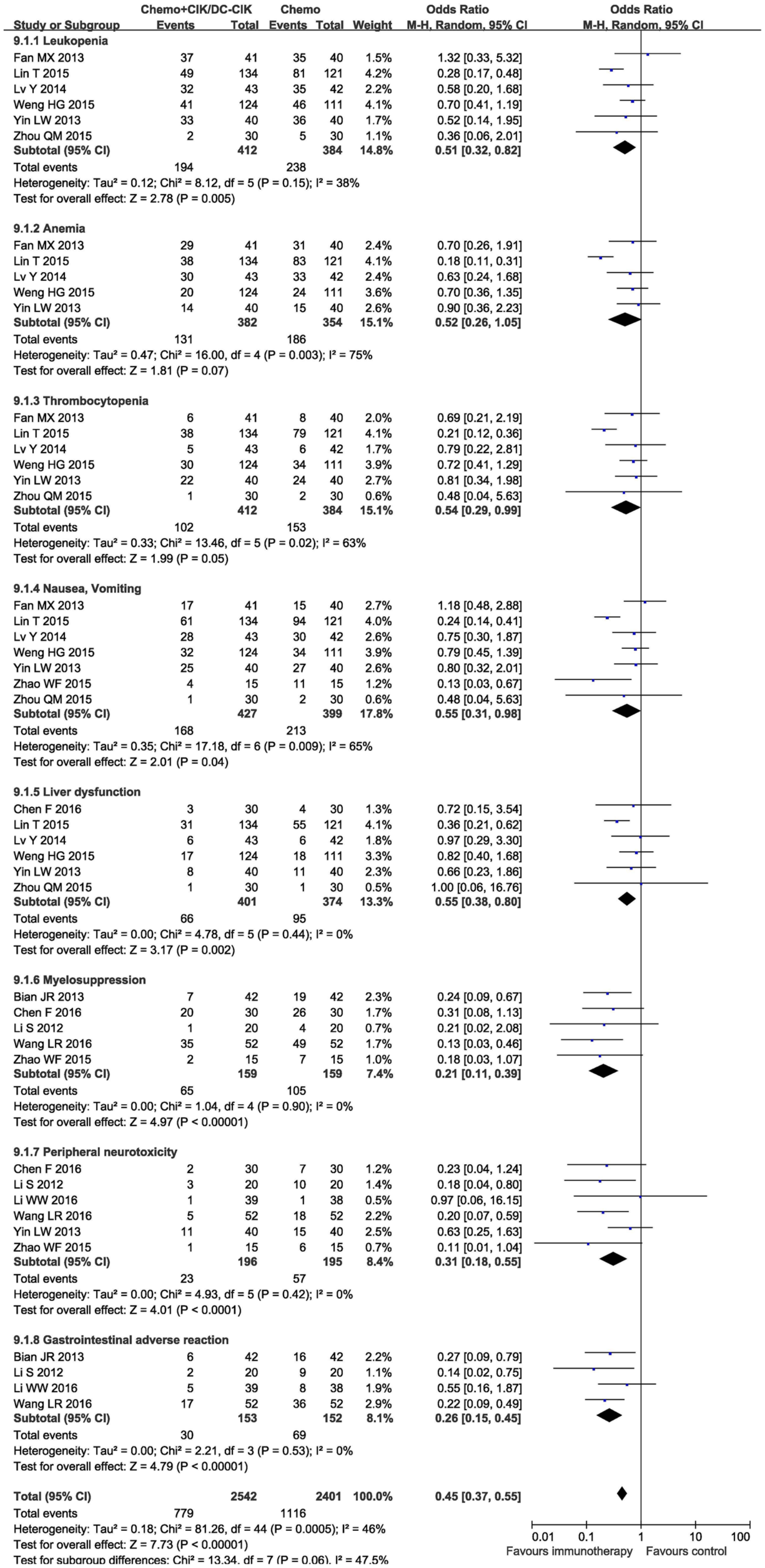

Supplementary Figure 4: Forest plot of the comparison of adverse effects. CI, confidence interval; Chemo, chemotherapy; CIK/DC-CIK, CIK/DC-CIK immunotherapy. The random effects meta-analysis model (Mantel–Haenszel method) was used.

Study or Subgroup	Chemo+CIK/DC-CIK Events	Chemo+CIK/DC-CIK Total	Chemo Events	Chemo Total	Weight	Odds Ratio M-H, Random, 95% CI
**10.1.1 Leukopenia I**						
Fan MX 2013	20	41	17	40	7.7%	1.29 [0.54, 3.10]
Lin T 2015	23	134	27	121	11.0%	0.72 [0.39, 1.34]
Weng HG 2015	10	124	7	111	6.5%	1.30 [0.48, 3.55]
Yin LW 2013	8	40	5	40	4.9%	1.75 [0.52, 5.90]
**Subtotal (95% CI)**		**339**		**312**	**30.0%**	**1.02 [0.67, 1.56]**
Total events	61		56			
Heterogeneity: Tau² = 0.00; Chi² = 2.46, df = 3 (P = 0.48); I² = 0%						
Test for overall effect: Z = 0.10 (P = 0.92)						
**10.1.2 Leukopenia II**						
Fan MX 2013	12	41	14	40	7.1%	0.77 [0.30, 1.96]
Lin T 2015	15	134	31	121	10.2%	0.37 [0.19, 0.72]
Weng HG 2015	20	124	16	111	9.6%	1.14 [0.56, 2.33]
Yin LW 2013	18	40	14	40	7.4%	1.52 [0.62, 3.74]
**Subtotal (95% CI)**		**339**		**312**	**34.3%**	**0.81 [0.42, 1.56]**
Total events	65		75			
Heterogeneity: Tau² = 0.27; Chi² = 8.04, df = 3 (P = 0.05); I² = 63%						
Test for overall effect: Z = 0.63 (P = 0.53)						
**10.1.3 Leukopenia III**						
Fan MX 2013	4	41	2	40	2.7%	2.05 [0.35, 11.90]
Lin T 2015	7	134	17	121	7.2%	0.34 [0.13, 0.84]
Weng HG 2015	7	124	17	111	7.2%	0.33 [0.13, 0.83]
Yin LW 2013	5	40	13	40	5.3%	0.30 [0.09, 0.93]
**Subtotal (95% CI)**		**339**		**312**	**22.5%**	**0.40 [0.21, 0.75]**
Total events	23		49			
Heterogeneity: Tau² = 0.09; Chi² = 3.88, df = 3 (P = 0.28); I² = 23%						
Test for overall effect: Z = 2.87 (P = 0.004)						
**10.1.4 Leukopenia IV**						
Fan MX 2013	1	41	2	40	1.5%	0.47 [0.04, 5.46]
Lin T 2015	4	134	6	121	4.5%	0.59 [0.16, 2.14]
Weng HG 2015	4	124	6	111	4.5%	0.58 [0.16, 2.12]
Yin LW 2013	2	40	4	40	2.7%	0.47 [0.08, 2.75]
**Subtotal (95% CI)**		**339**		**312**	**13.1%**	**0.55 [0.26, 1.19]**
Total events	11		18			
Heterogeneity: Tau² = 0.00; Chi² = 0.06, df = 3 (P = 1.00); I² = 0%						
Test for overall effect: Z = 1.52 (P = 0.13)						
**Total (95% CI)**		**1356**		**1248**	**100.0%**	**0.72 [0.53, 0.98]**
Total events	160		198			
Heterogeneity: Tau² = 0.13; Chi² = 22.77, df = 15 (P = 0.09); I² = 34%						
Test for overall effect: Z = 2.09 (P = 0.04)						
Test for subgroup differences: Chi² = 6.60, df = 3 (P = 0.09), I² = 54.5%						

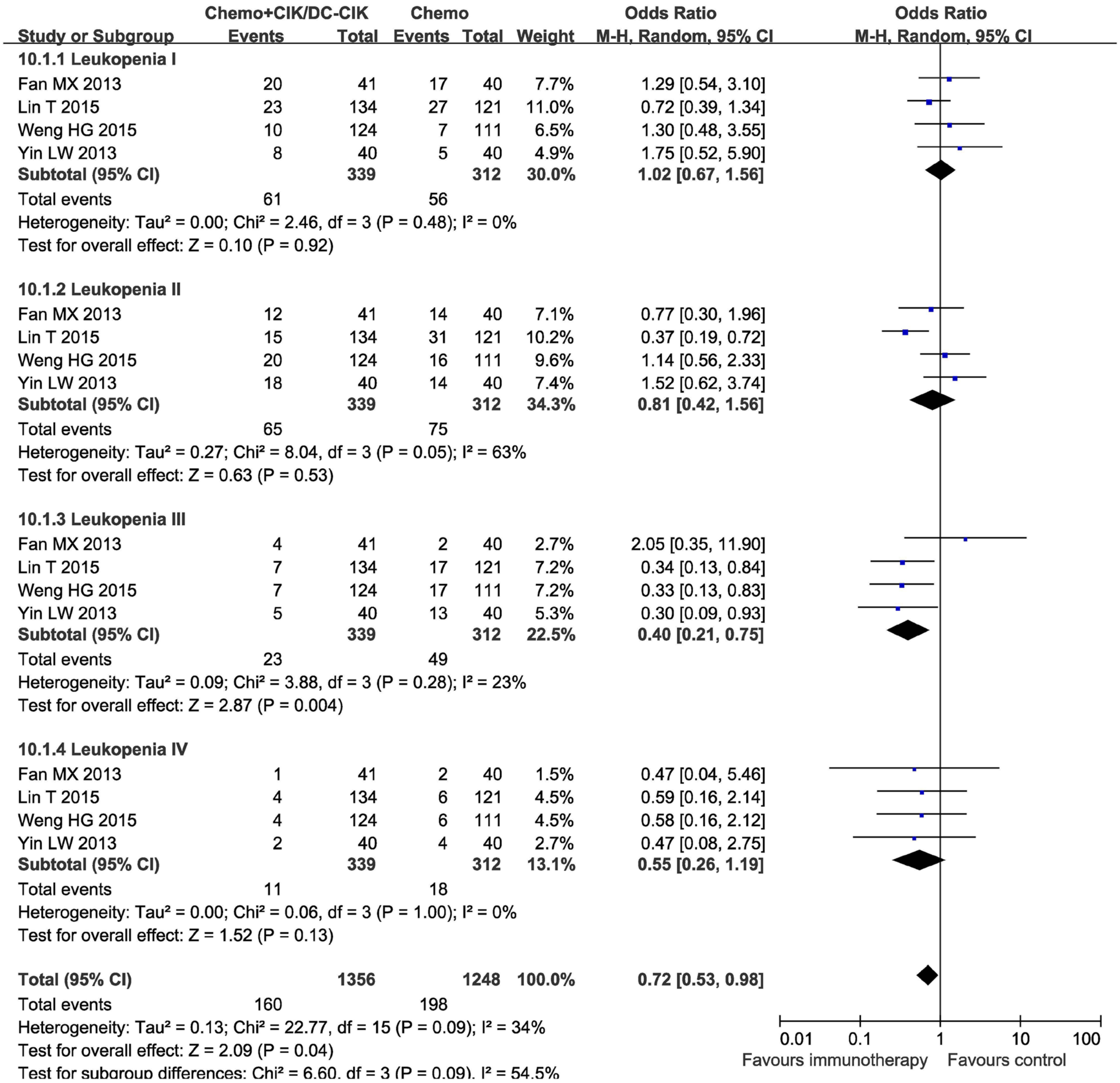

Supplementary Figure 5: Forest plot of the comparison of all-grade leucopenia. CI, confidence interval; Chemo, chemotherapy; CIK/DC-CIK, CIK/DC-CIK immunotherapy. The random effects meta-analysis model (Mantel–Haenszel method) was used.

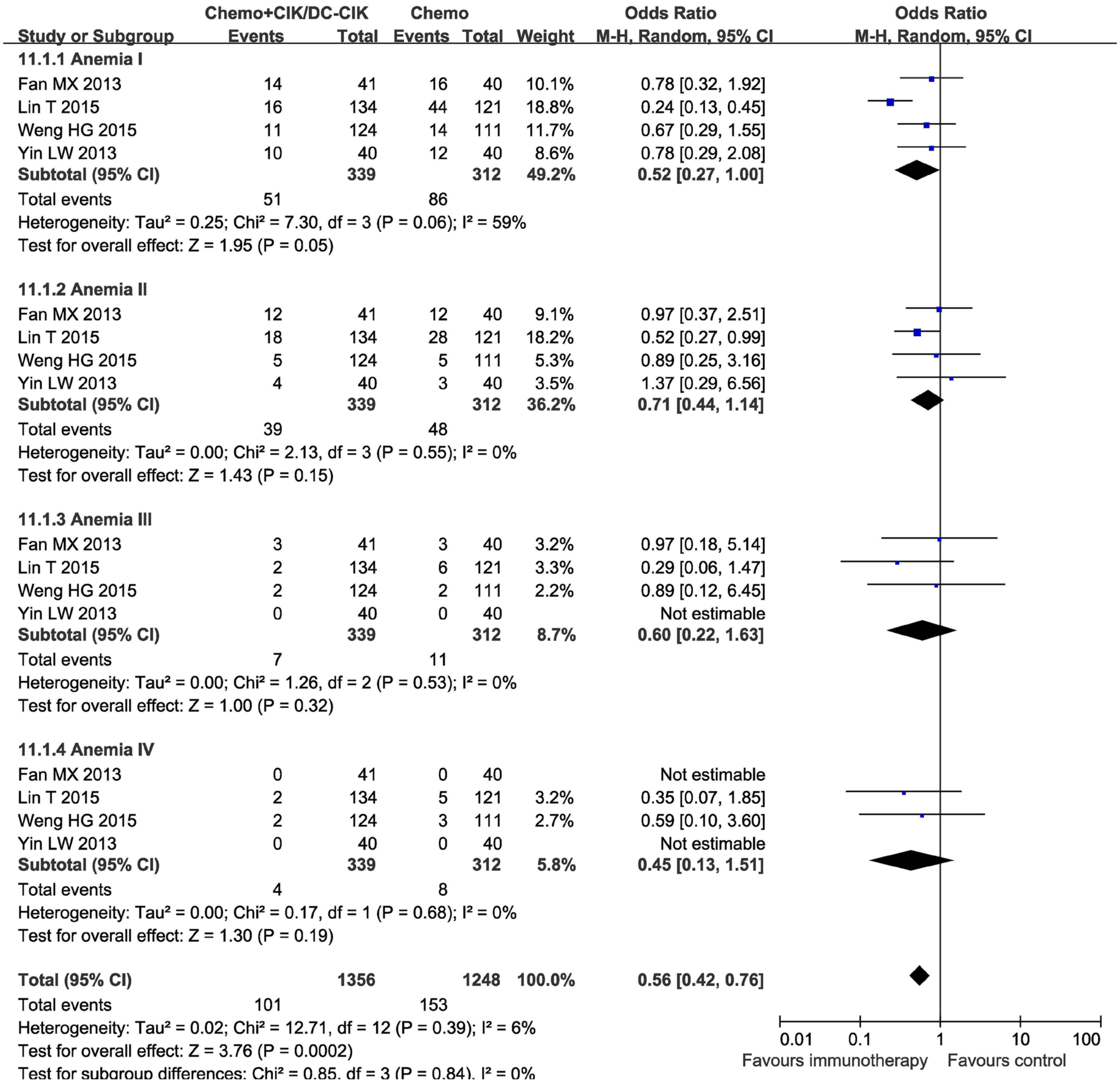

Supplementary Figure 6: Forest plot of the comparison of all-grade anemia. CI, confidence interval; Chemo, chemotherapy; CIK/DC-CIK, CIK/DC-CIK immunotherapy. The random effects meta-analysis model (Mantel–Haenszel method) was used.

Study or Subgroup	Chemo+CIK/DC-CIK Events	Chemo+CIK/DC-CIK Total	Chemo Events	Chemo Total	Weight	Odds Ratio M-H, Random, 95% CI
**12.1.1 Thrombocytopenia I**						
Fan MX 2013	4	41	5	40	5.2%	0.76 [0.19, 3.05]
Lin T 2015	15	134	29	121	15.3%	0.40 [0.20, 0.79]
Weng HG 2015	15	124	15	111	13.1%	0.88 [0.41, 1.90]
Yin LW 2013	13	40	12	40	9.8%	1.12 [0.44, 2.89]
**Subtotal (95% CI)**		**339**		**312**	**43.4%**	**0.69 [0.42, 1.13]**
Total events	47		61			
Heterogeneity: Tau² = 0.06; Chi² = 3.88, df = 3 (P = 0.27); I² = 23%						
Test for overall effect: Z = 1.47 (P = 0.14)						
**12.1.2 Thrombocytopenia II**						
Fan MX 2013	2	41	2	40	2.7%	0.97 [0.13, 7.27]
Lin T 2015	18	134	37	121	16.7%	0.35 [0.19, 0.66]
Weng HG 2015	10	124	8	111	9.5%	1.13 [0.43, 2.97]
Yin LW 2013	8	40	6	40	7.1%	1.42 [0.44, 4.53]
**Subtotal (95% CI)**		**339**		**312**	**36.0%**	**0.76 [0.35, 1.69]**
Total events	38		53			
Heterogeneity: Tau² = 0.34; Chi² = 6.71, df = 3 (P = 0.08); I² = 55%						
Test for overall effect: Z = 0.67 (P = 0.50)						
**12.1.3 Thrombocytopenia III**						
Fan MX 2013	0	41	1	40	1.1%	0.32 [0.01, 8.02]
Lin T 2015	3	134	11	121	5.9%	0.23 [0.06, 0.84]
Weng HG 2015	3	124	9	111	5.7%	0.28 [0.07, 1.07]
Yin LW 2013	1	40	6	40	2.4%	0.15 [0.02, 1.27]
**Subtotal (95% CI)**		**339**		**312**	**15.0%**	**0.24 [0.10, 0.54]**
Total events	7		27			
Heterogeneity: Tau² = 0.00; Chi² = 0.29, df = 3 (P = 0.96); I² = 0%						
Test for overall effect: Z = 3.41 (P = 0.0006)						
**12.1.4 Thrombocytopenia IV**						
Fan MX 2013	0	41	0	40		Not estimable
Lin T 2015	2	134	2	121	2.8%	0.90 [0.13, 6.50]
Weng HG 2015	2	124	2	111	2.8%	0.89 [0.12, 6.45]
Yin LW 2013	0	40	0	40		Not estimable
**Subtotal (95% CI)**		**339**		**312**	**5.6%**	**0.90 [0.22, 3.63]**
Total events	4		4			
Heterogeneity: Tau² = 0.00; Chi² = 0.00, df = 1 (P = 0.99); I² = 0%						
Test for overall effect: Z = 0.15 (P = 0.88)						
**Total (95% CI)**		**1356**		**1248**	**100.0%**	**0.59 [0.42, 0.84]**
Total events	96		145			
Heterogeneity: Tau² = 0.08; Chi² = 16.26, df = 13 (P = 0.24); I² = 20%						
Test for overall effect: Z = 2.98 (P = 0.003)						
Test for subgroup differences: Chi² = 5.79, df = 3 (P = 0.12), I² = 48.1%						

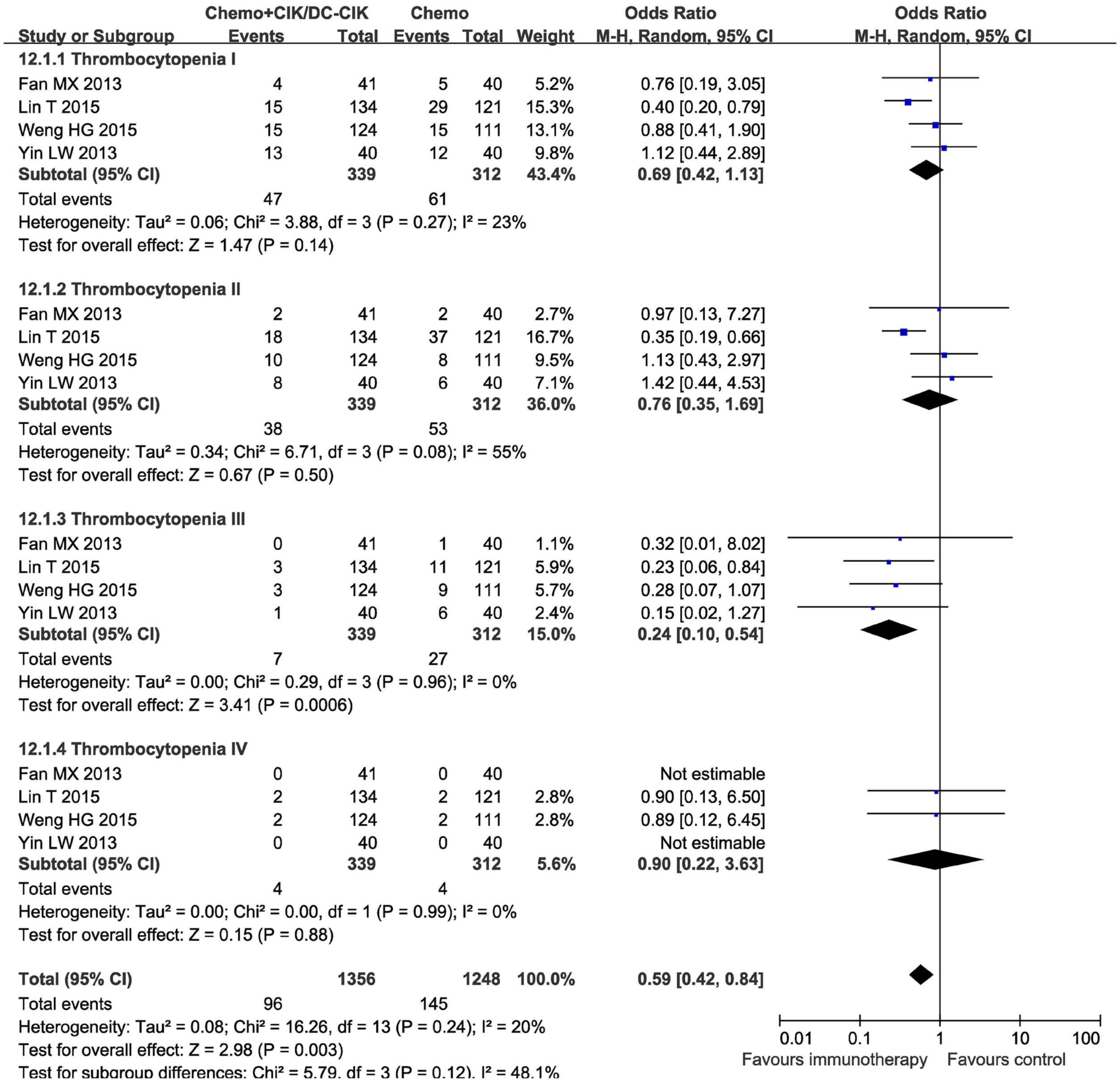

Supplementary Figure 7: Forest plot of the comparison of all-grade thrombocytopenia. CI, confidence interval; Chemo, chemotherapy; CIK/DC-CIK, CIK/DC-CIK immunotherapy. The random effects meta-analysis model (Mantel–Haenszel method) was used.

Study or Subgroup	Chemo+CIK/DC-CIK Events	Chemo+CIK/DC-CIK Total	Chemo Events	Chemo Total	Weight	Odds Ratio M-H, Random, 95% CI
**13.1.1 Nausea, Vomiting I**						
Fan MX 2013	9	41	8	40	6.5%	1.13 [0.39, 3.28]
Lin T 2015	24	134	39	121	18.2%	0.46 [0.26, 0.82]
Weng HG 2015	14	124	8	111	8.7%	1.64 [0.66, 4.07]
Yin LW 2013	12	40	7	40	6.6%	2.02 [0.70, 5.83]
**Subtotal (95% CI)**		**339**		**312**	**40.0%**	**1.06 [0.49, 2.29]**
Total events	59		62			
Heterogeneity: Tau² = 0.40; Chi² = 9.12, df = 3 (P = 0.03); I² = 67%						
Test for overall effect: Z = 0.15 (P = 0.88)						
**13.1.2 Nausea, Vomiting II**						
Fan MX 2013	5	41	5	40	4.3%	0.97 [0.26, 3.65]
Lin T 2015	33	134	46	121	20.6%	0.53 [0.31, 0.91]
Weng HG 2015	14	124	18	111	12.1%	0.66 [0.31, 1.39]
Yin LW 2013	12	40	16	40	8.4%	0.64 [0.25, 1.62]
**Subtotal (95% CI)**		**339**		**312**	**45.5%**	**0.61 [0.42, 0.89]**
Total events	64		85			
Heterogeneity: Tau² = 0.00; Chi² = 0.77, df = 3 (P = 0.86); I² = 0%						
Test for overall effect: Z = 2.56 (P = 0.01)						
**13.1.3 Nausea, Vomiting III**						
Fan MX 2013	3	41	2	40	2.3%	1.50 [0.24, 9.49]
Lin T 2015	3	134	7	121	4.0%	0.37 [0.09, 1.48]
Weng HG 2015	3	124	6	111	3.9%	0.43 [0.11, 1.78]
Yin LW 2013	1	40	4	40	1.6%	0.23 [0.02, 2.16]
**Subtotal (95% CI)**		**339**		**312**	**11.8%**	**0.48 [0.21, 1.08]**
Total events	10		19			
Heterogeneity: Tau² = 0.00; Chi² = 2.03, df = 3 (P = 0.57); I² = 0%						
Test for overall effect: Z = 1.77 (P = 0.08)						
**13.1.4 Nausea, Vomiting IV**						
Fan MX 2013	0	41	0	40		Not estimable
Lin T 2015	1	134	2	121	1.4%	0.45 [0.04, 5.00]
Weng HG 2015	1	124	2	111	1.4%	0.44 [0.04, 4.95]
Yin LW 2013	0	40	0	40		Not estimable
**Subtotal (95% CI)**		**339**		**312**	**2.7%**	**0.45 [0.08, 2.45]**
Total events	2		4			
Heterogeneity: Tau² = 0.00; Chi² = 0.00, df = 1 (P = 1.00); I² = 0%						
Test for overall effect: Z = 0.93 (P = 0.35)						
**Total (95% CI)**		**1356**		**1248**	**100.0%**	**0.69 [0.52, 0.92]**
Total events	135		170			
Heterogeneity: Tau² = 0.03; Chi² = 14.30, df = 13 (P = 0.35); I² = 9%						
Test for overall effect: Z = 2.57 (P = 0.01)						
Test for subgroup differences: Chi² = 2.38, df = 3 (P = 0.50), I² = 0%						

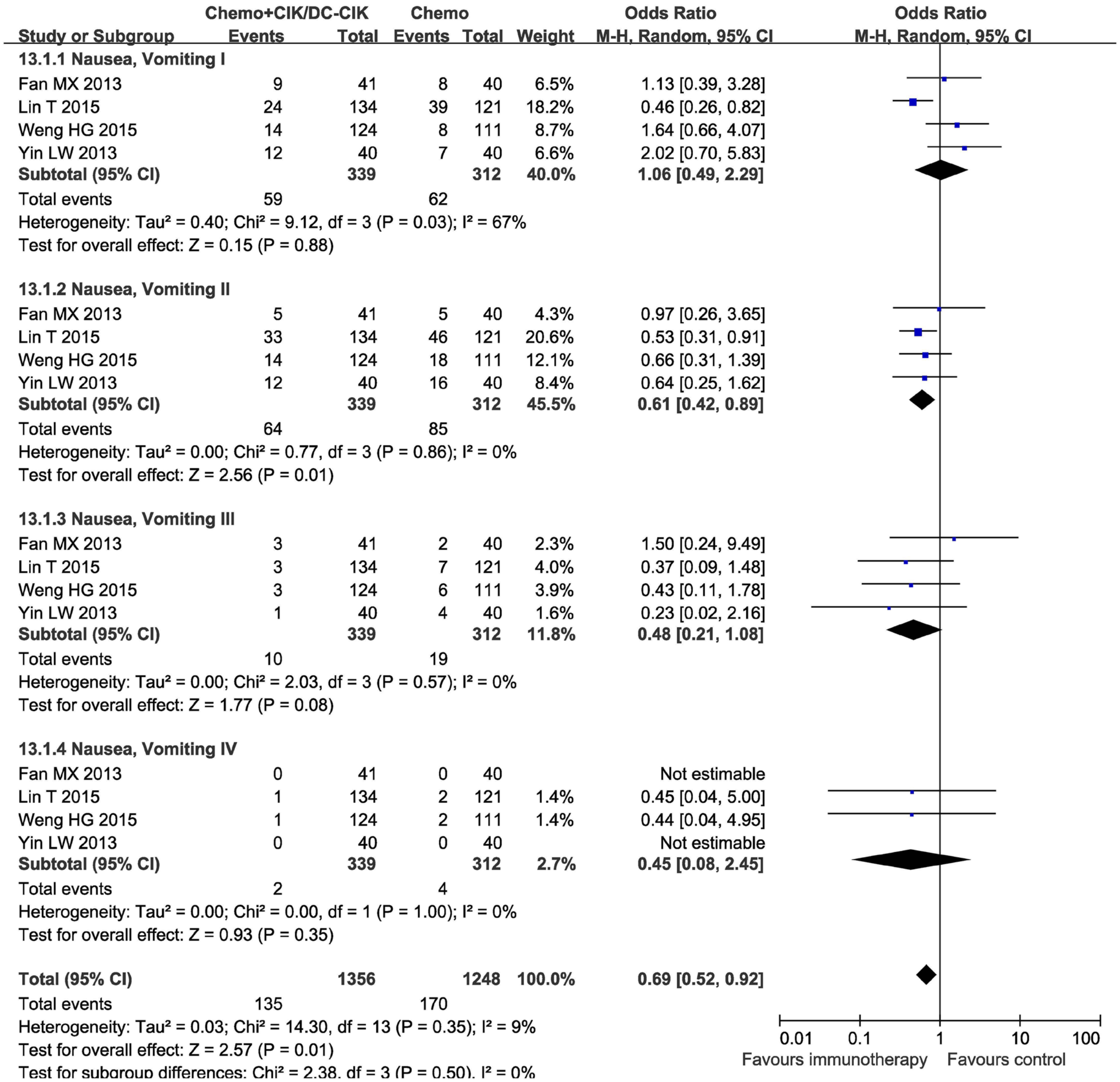

Supplementary Figure 8: Forest plot of the comparison of all-grade nausea and vomiting. CI, confidence interval; Chemo, chemotherapy; CIK/DC-CIK, CIK/DC-CIK immunotherapy. The random effects meta-analysis model (Mantel–Haenszel method) was used.

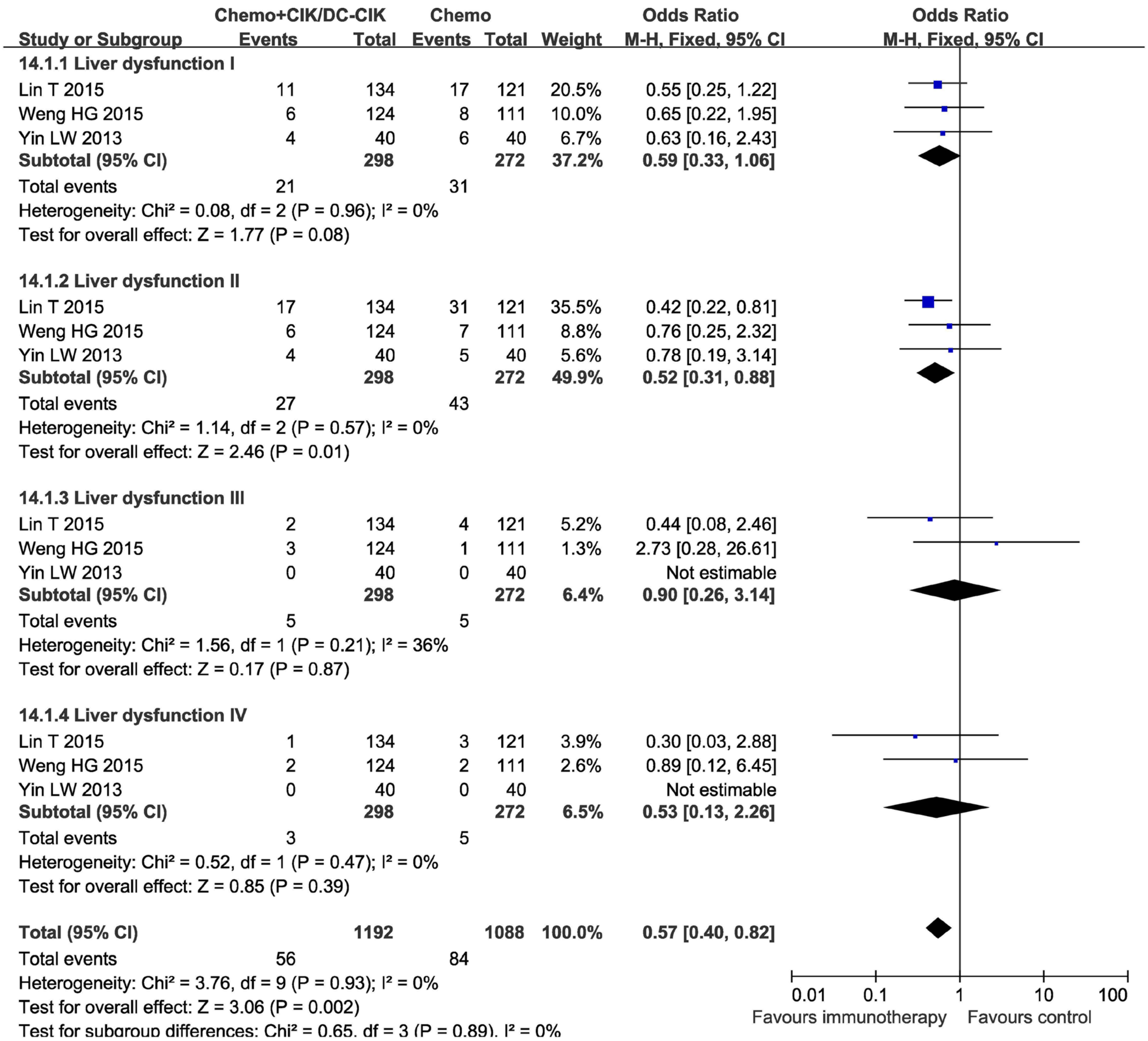

Supplementary Figure 9: Forest plot of the comparison of all-grade liver dysfunction. CI, confidence interval; Chemo, chemotherapy; CIK/DC-CIK, CIK/DC-CIK immunotherapy. The fixed-effects meta-analysis model (Mantel–Haenszel method) was used.

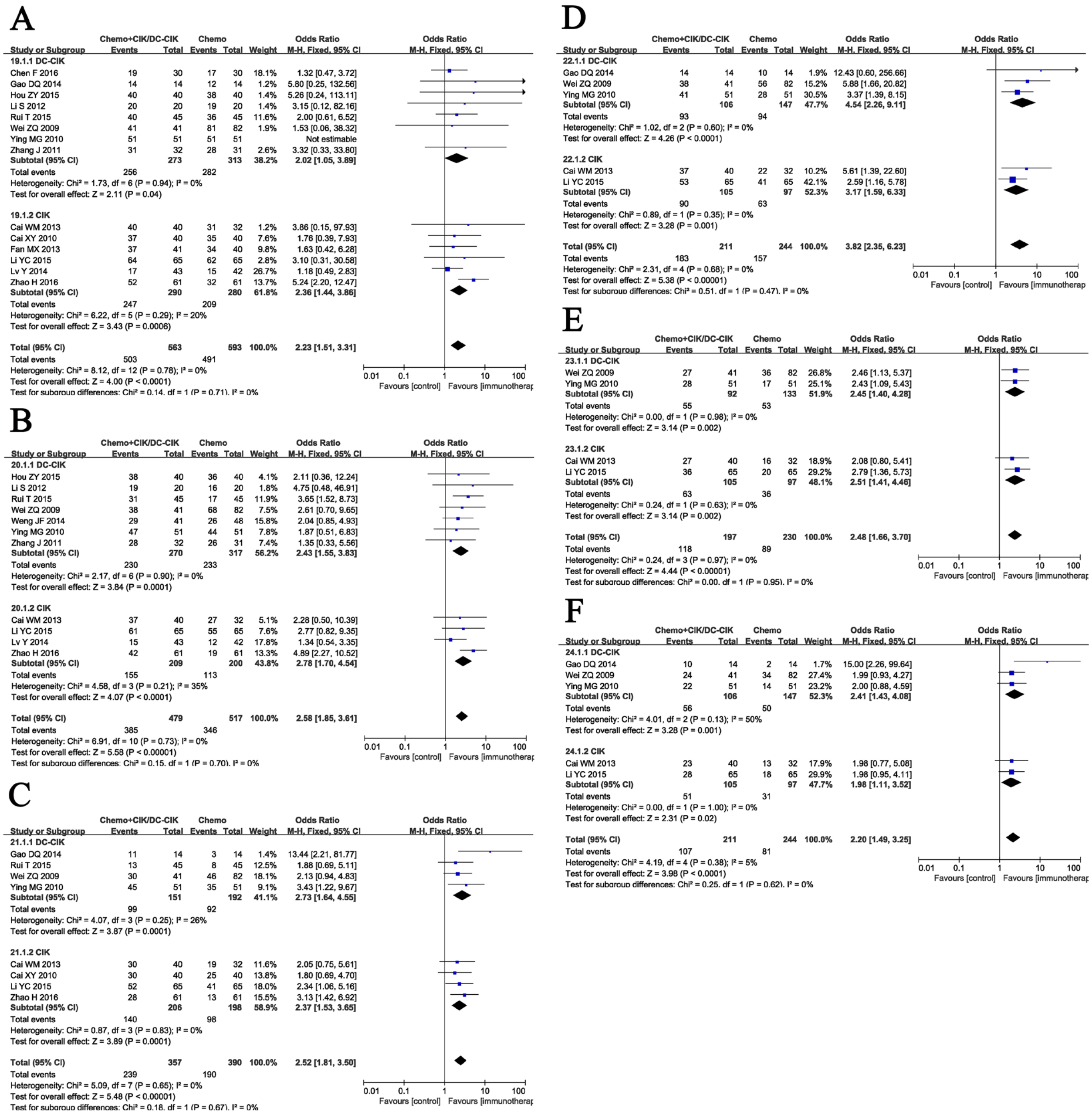

Supplementary Figure 10: Forest plot of overall survival (OS) and disease free survival (DFS) in Chemo-DC-CIK and Chemo-CIK subgroups. 1-year OS (**A**); 2-year OS (**B**); 3-year OS (**C**); 1-year DFS (**D**); 2-year DFS (**E**); 3-year DFS (**F**). CI, confidence interval; Chemo-CIK, chemotherapy combined with CIK immunotherapy; Chemo-DC-CIK, chemotherapy combined with DC-CIK immunotherapy. The fixed-effects meta-analysis model (Mantel–Haenszel method) was used.

A

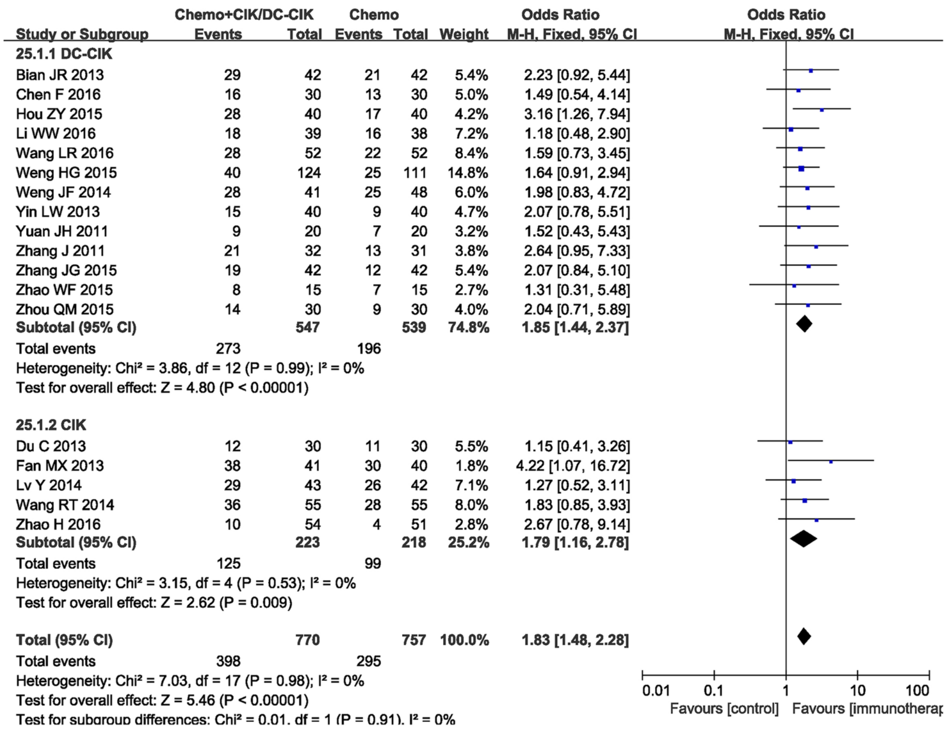

B

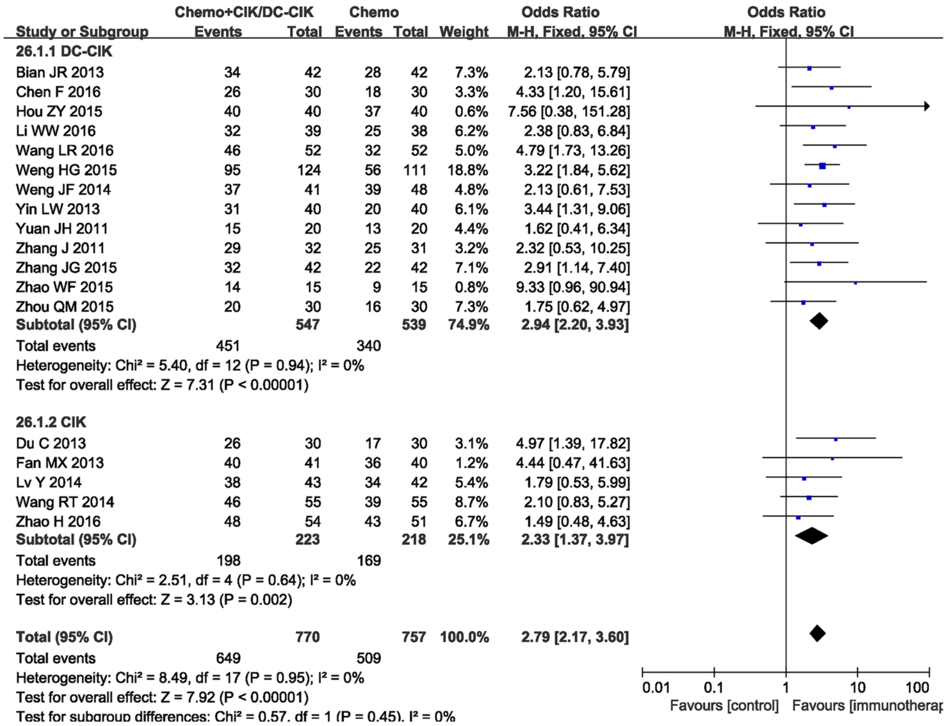

Supplementary Figure 11: Forest plot of overall response rate (ORR) and disease control rate (DCR) in Chemo-DC-CIK and Chemo-CIK subgroups. CI, confidence interval; Chemo-CIK, chemotherapy combined with CIK immunotherapy; Chemo-DC-CIK, chemotherapy combined with DC-CIK immunotherapy. The fixed-effects meta-analysis model (Mantel– Haenszel method) was used.

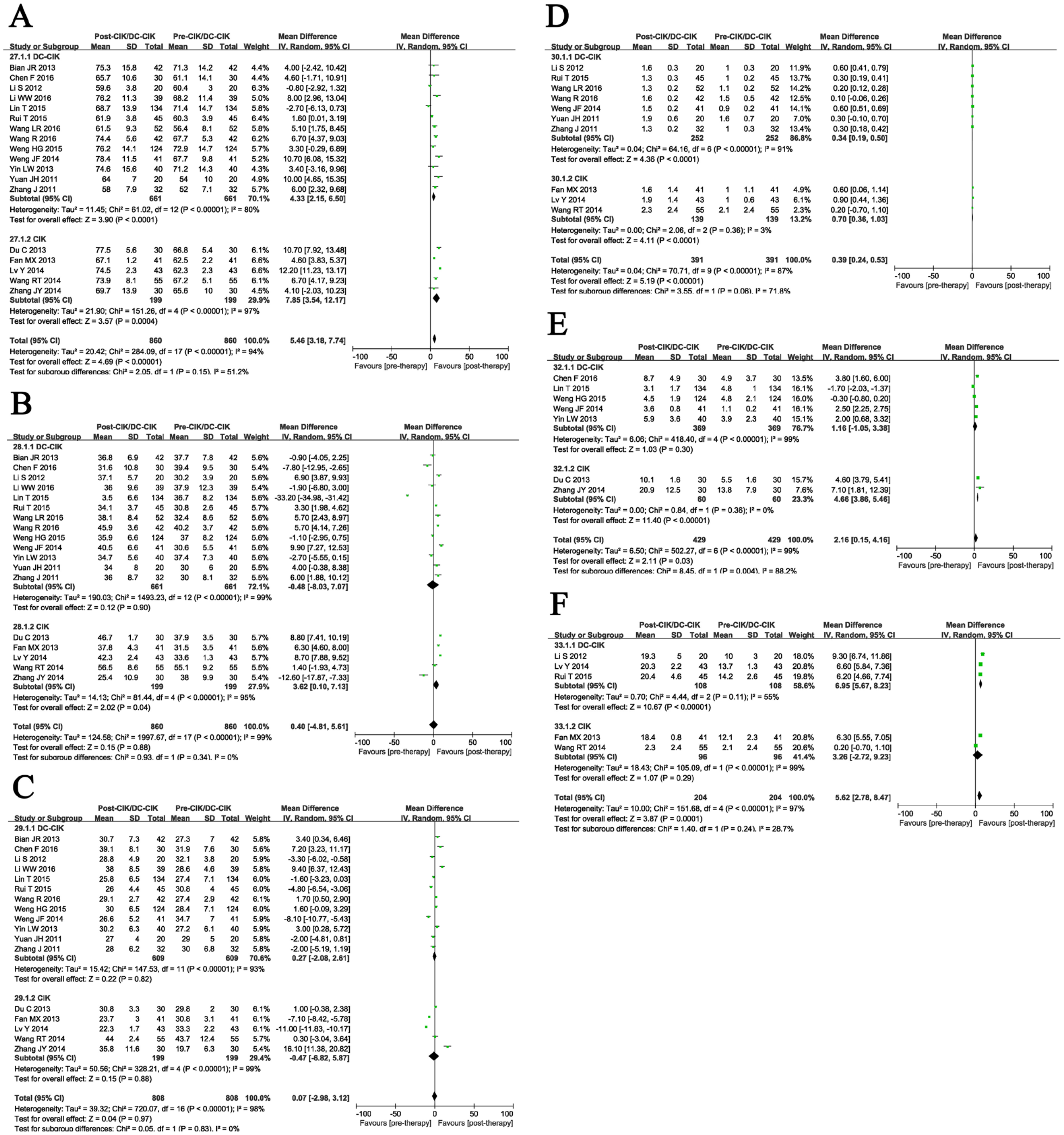

Supplementary Figure 12: Forest plot of immunophenotype in Chemo-DC-CIK and Chemo-CIK subgroups. $CD3^{+}$ (**A**); $CD4^{+}$ (**B**); $CD8^{+}$ (**C**) $CD4^{+}/CD8^{+}$ (**D**); $CD3^{+}CD56^{+}$ (**E**); $CD16^{+}CD56^{+}$ (**F**). CI, confidence interval; Chemo-CIK, chemotherapy combined with CIK immunotherapy; Chemo-DC-CIK, chemotherapy combined with DC-CIK immunotherapy. The random effects meta-analysis model (Mantel–Haenszel method) was used.

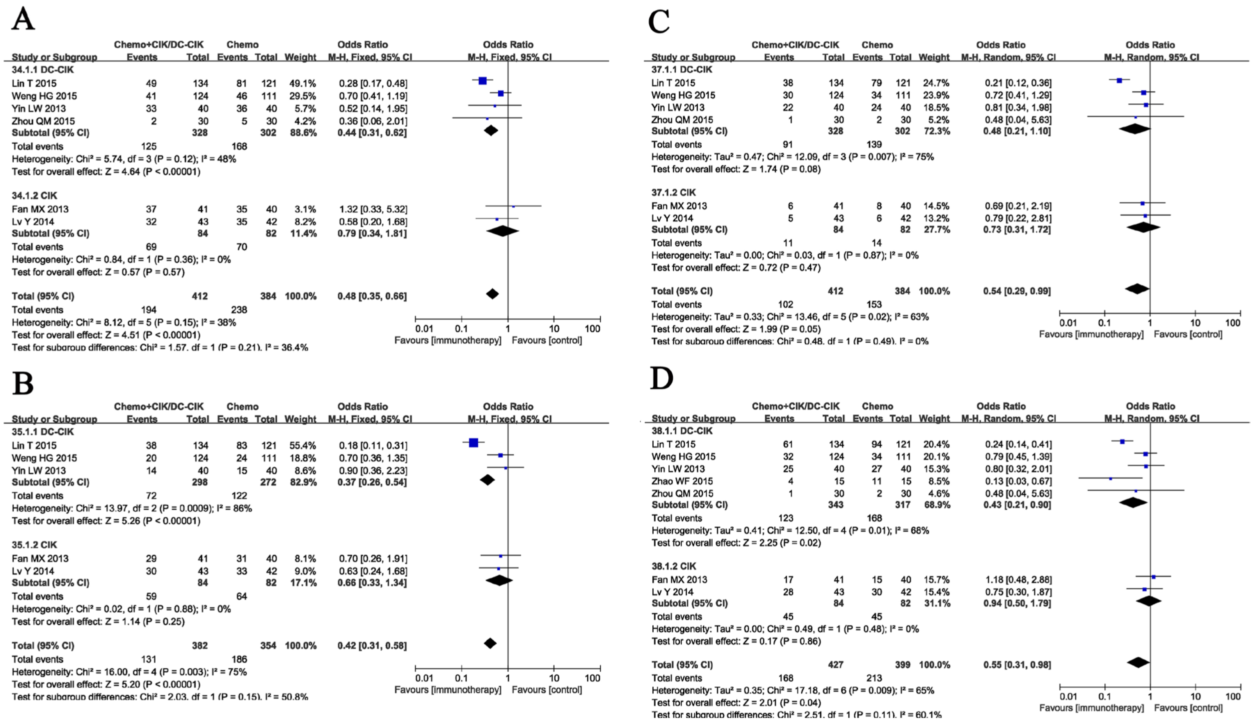

Supplementary Figure 13: Forest plot of adverse effects in Chemo-DC-CIK and Chemo-CIK subgroups. Leucopenia (**A**); Anemia (**B**); Thrombocytopenia (**C**) Nausea and vomiting (**D**). CI, confidence interval; Chemo-CIK, chemotherapy combined with CIK immunotherapy; Chemo-DC-CIK, chemotherapy combined with DC-CIK immunotherapy. The random effects meta-analysis model (Mantel–Haenszel method) was used.

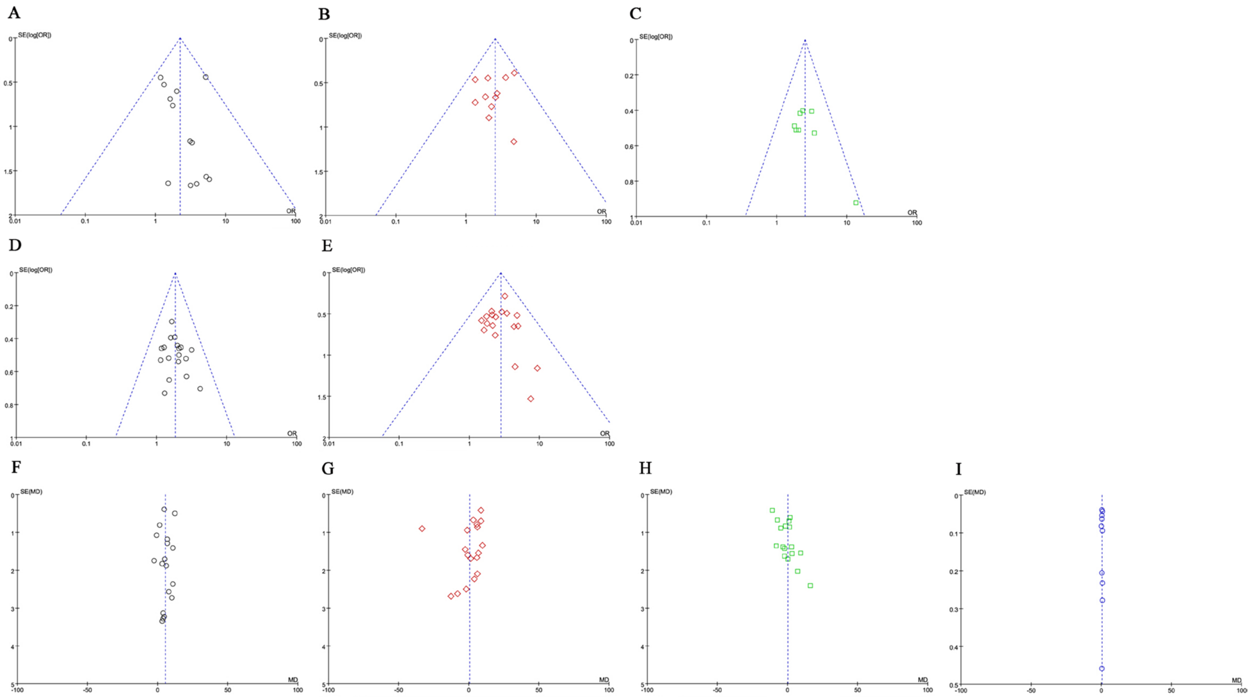

Supplementary Figure 14: Funnel plot of each meta-analysis. 1-year OS (**A**); 2-year OS (**B**); 3-year OS (**C**); ORR (**D**); DCR (**E**); CD3$^+$ (**F**); CD4$^+$ (**G**); CD8$^+$ (**H**) CD4$^+$/CD8$^+$ (**I**). Parameters discussed in over 8 papers were conducted bias analyses.

Supplementary Table 1: Clinical information from the eligible trials used in the meta-analysis. See Supplementary_Table_1
