# Supplementary material for: Cytokine-induced killer cells/dendritic cells-cytokine induced killer cells immunotherapy combined with chemotherapy for treatment of colorectal cancer in China: a meta-analysis of 29 trials involving 2,610 patients
Source: Oncotarget. 2017 Mar 29;8(28):45164–77. doi: 10.18632/oncotarget.16665 (PMC5542175; doi:10.18632/oncotarget.16665)
Supplement: Supplementary file 2 [file oncotarget-08-45164-s002.doc]

**Table 1.** Clinical information from the eligible trials used in the meta-analysis. The table summarizes patients’ basic information regarding the tumor stage, treatment regimens, cases, age, and details of the immunotherapy (culture conditions, cell does and the treatment courses). CT, chemotherapy; Con, control group; Exp, experimental group; ND: non determined; cap: capecitabine; oxa: oxaliplatin; bev: bevacizumab；leu: leucovorin; cf: calcium folinate; fu: fluorouracil; iri: irinotecan; cis: cisplatin; Xelox: cap+oxa; Folfox: oxa+cf+5-fu; Folfiri:cf+iri+5-fu; a: co-injection of DC with CIK; b: DC cultivated with CIK before injection.

| Authors and year | Tumor  types | stage | Exp regimens  CT regimens | Patients Con/Exp | Age (year) | | Culture conditions | Cell dose (cycles) |
| --- | --- | --- | --- | --- | --- | --- | --- | --- |
|  |  |  |  |  | Con | Exp |  |  |
| Bian [29]  2013 | colorectal cancer | ND | CT-DC-CIK^b^  Folfox | 42/42 | 62.2±4.9  (mean) | 62.7±4.9  (mean) | IFN-γ, IL-2, CD28 (CIK)  GM-CSF, IL-4, TNF-α, PGE-2,  IL-1β (DC) | ND (2 cycles) |
| Cai [30]  2013 | rectal cancer | I-III | CT-CIK  Folfox/Xelox /cap/cf+5-fu | 32/40 | 65  (Median) | 66  (Median) | IFN-γ, IL-1α, IL-2, CD3 (CIK) | ND (1 cycle) |
| Cai [31]  2010 | rectal cancer | II-III | CT-CIK  Xelox | 40/40 | 44.5  (Median) | 46.7  (Median) | IFN-γ, IL-1α, IL-2, CD3 (CIK) | ND (3 cycles) |
| Chen [32]  2016 | colon cancer | III-IV | CT-DC-CIK^b^  Xelox+bev | 30/30 | 50  (Median) | 52  (Median) | IFN-γ, IL-2, CD3 (CIK)  GM-CSF, IL-4, IFN-γ (DC) | ND (2 cycles) |
| Du [33] 2013 | colorectal cancer | Kps≥70 | CT-CIK  Xelox | 30/30 | ≥60 n=12  (range) | ≥60 n=10  (range) | IFN-γ, IL-2, CD3 (CIK) | 1.5×10^10^/cycle  (ND) |
| Fan [34]  2013 | rectal cancer | Kps≥60 | CT-CIK  Folfox/Xelox | 40/41 | ≥60 n=26  (range) | ≥60 n=23  (range) | IFN-γ, IL-2, CD3 (CIK) | 4.5-18.9×10^9^/cycle  (ND) |
| Gao [18]  2014 | colorectal cancer | I-IV | CT-DC-CIK^a^  ND | 14/14 | 61.6±12.7  (mean) | 64.5±12.8  (mean) | IFN-γ, IL-1α, IL-2, CD3 (CIK)  GM-CSF, IL-4 (DC) | ND (2 cycles) |
| Hou [35]  2015 | colon cancer | II-III | CT-DC-CIK^b^  Folfox | 40/40 | 57.4±3.0  (mean) | 56.0±2.5  (mean) | ND | ND (2 cycles) |
| Li [36]  2012 | colon cancer | II-III | CT-DC-CIK^b^  Folfox | 20/20 | 57.5  (mean) | 54.5  (mean) | IFN-γ, IL-2, CD3 (CIK)  GM-CSF, IL-4, IFN-γ (DC) | ND (2 cycles) |
| Li [37]  2016 | colorectal cancer | Ps≤2 | CT-DC-CIK^b^  Folfox | 38/39 | 51.6 ±13.0  (mean) | 53.4±13.6  (mean) | ND | ND (ND) |
| Li [38]  2015 | colorectal cancer | II-III | CT-CIK  Folfox/Xelox/Cap | 65/65 | 58±12  (mean) | 57±13  (mean) | IFN-γ, IL-1α, IL-2, CD3 (CIK) | about 5×10^9^/cycle  (≥4 cycles) |
| Lin [39]  2016 | colorectal cancer | Kps>60 | CT-DC-CIK^a^  Folfox/Xelox | 121/134 | ≥60 n=53  (range) | ≥60 n=55  (range) | IFN-γ, IL-1α, IL-2, CD3 (CIK)  GM-CSF, IL-4, TNF-α (DC) | 1×10^7^/cycle (DC)  1×10^9^/cycle (CIK)  (5 cycles) |
| Lv [40] 2014 | colorectal cancer | Kps>70 | CT-CIK  Folfox | 42/43 | ≥60 n=26  (range) | ≥60 n=23  (range) | IFN-γ, IL-2, CD3 (CIK) | (4.5-19)×10^9^/cycle (ND) |
| Rui [41]  2015 | colorectal cancer | IV | CT-DC-CIK^a^  Folfox | 45/45 | 58.6±12.6  (mean) | 63.3±10.1  (mean) | IFN-γ, IL-1α, IL-2, CD3 (CIK)  GM-CSF, IL-4 (DC) | ND (≥3 cycles) |
| Wang [42]  2016 | colon cancer | III-IV | CT-DC-CIK^a^  Xelox | 52/52 | 50.2±3.7 (mean) | 50.2±3.7 (mean) | IFN-γ, IL-1α, IL-2, CD3 (CIK) | ND (2 cycles) |
| Wang [43]  2016 | colorectal cancer | III-IV | CT-DC-CIK^b^  Folfox | 42/42 | 54  (Median) | 56  (Median) | ND | ND (2-3 cycles) |
| Wang [44] 2014 | colorectal cancer | ND | CT-CIK  oxa+leu+5-fu | 55/55 | 64  (mean) | 64  (mean) | IFN-γ, IL-2, IL-15, CD3 (CIK) | 3-9×10^9^/cycle,  (5 cycles) |
| Wei [45]  2009 | rectal cancer | I-III | CT-DC-CIK^b^  Folfox/Xelox | 82/41 | 55.5  (Median) | 54  (Median) | IFN-γ, IL-1α, IL-2, CD3 (CIK)  GM-CSF, IL-4, TNF-α (DC) | ≥8×10^10^/cycle  (1-6 cycles) |
| Weng [46]  2015 | colorectal cancer | III-IV | CT-DC-CIK^b^  Folfox | 111/124 | 55  (Median) | 59  (Median) | ND | ND (3 cycles) |
| Weng [47]  2014 | rectal cancer | IV | CT-DC-CIK^a^  Folfox | 48/41 | 53  (mean) | 52  (mean) | IFN-γ, IL-1α, IL-2, CD3 (CIK)  IFN-γ, LPS (DC) | 3×10^7-9^/cycle (DC)  5×10^9^/cycle (CIK)  (2 cycles) |
| Yin [48]  2013 | rectal cancer | Ps≤2 | CT-DC-CIK^a^  Folfox/Folfiri | 40/40 | 63  (Median) | 62  (Median) | IFN-γ, IL-2, CD3 (CIK)  GM-CSF, IL-4, TNF-α (DC) | 3-8×10^8^/cycle (DC)  1-4×10^9^/cycle (CIK)  (≥3 cycles) |
| Ying [49]  2010 | colorectal cancer | I-III | CT-DC-CIK^b^  Folfox/Xelox/ cf+5-fu/cap | 51/51 | 54  (Median) | 59  (Median) | IFN-γ, IL-1α, IL-2, CD3 (CIK)  GM-CSF, IL-4, TNF-α (DC) | ≥8×10^10^/cycle  (1-6 cycles) |
| Yuan [50]  2011 | colorectal cancer | Kps≥60 | CT-DC-CIK^a^  Folfox | 21/21 | 58  (Median) | 60  (Median) | IFN-γ, IL-2, IL-1α, CD3 (CIK)  GM-CSF, IL-4, IFN-γ, TNF-α (DC) | >1×10^6^ (DC)  >1×10^10^ (CIK)  (4 cycles) |
| Zhang [51]  2011 | colon cancer | II-IV | CT-DC-CIK^a^  Folfox/Folfiri | 31/32 | ND | ND | IFN-γ, IL-2, IL-1α, CD3 (CIK)  GM-CSF, IL-4, TNF-α (DC) | >1×10^9^/cycle  (ND) |
| Zhang [52]  2015 | colorectal cancer | IV | CT-DC-CIK^b^  Folfox/Xelox/cap | 42/42 | >60 n=27  (range) | >60 n=26  (range) | IFN-γ, IL-2, CD3 (CIK)  GM-CSF, IL-4, IL-1β, PGE-2, TNF-α (DC) | 6×10^9^/cycle (ND) |
| Zhang [53]  2014 | colorectal cancer | I-IV | CT-CIK  Folfox/Xelox | 30/30 | ≥60 n=13  (range) | ≥60 n=18  (range) | IFN-γ, IL-2, CD3 (CIK) | ND (≥1 cycle) |
| Zhao [5]  2016 | colorectal cancer | Kps>70 | CT-CIK  Folfox | 61/61 | 60  (Median) | 58  (Median) | IFN-γ, IL-1α, IL-2, CD3 (CIK) | about1×10^10^/cycle  (≥1 cycle) |
| Zhao [54]  2015 | colon cancer | IV | CT-DC-CIK^b^  Xelox+bev | 15/15 | 53.5±4.9 (mean) | 52.5±5.1 (mean) | IFN-γ, IL-2, CD3 (CIK)  GM-CSF, IL-4, IFN-γ (DC) | ND (2 cycles) |
| Zhou [55]  2015 | colorectal cancer | III-IV | CT-DC-CIK^b^  Folfox | 30/30 | 65.1±4.5 (mean) | 64.1±4.6 (mean) | ND | ND (2 cycles) |
